# IPr^#^ Complexes—Highly-Hindered, Sterically-Bulky Cu(I) and Ag(I) N-Heterocyclic Carbenes: Synthesis, Characterization, and Reactivity

**DOI:** 10.1021/acs.organomet.4c00333

**Published:** 2024-09-21

**Authors:** Greta Utecht-Jarzyńska, Szymon Jarzyński, Md Mahbubur Rahman, Guangrong Meng, Roger Lalancette, Roman Szostak, Michal Szostak

**Affiliations:** † Department of Chemistry, Rutgers University, 73 Warren Street, Newark, New Jersey 07102, United States; ‡ Faculty of Chemistry, University of Lodz, Tamka 12, 91-403 Łódź, Poland; § Department of Chemistry, Wroclaw University, F. Joliot-Curie 14, Wroclaw 50-383, Poland

## Abstract

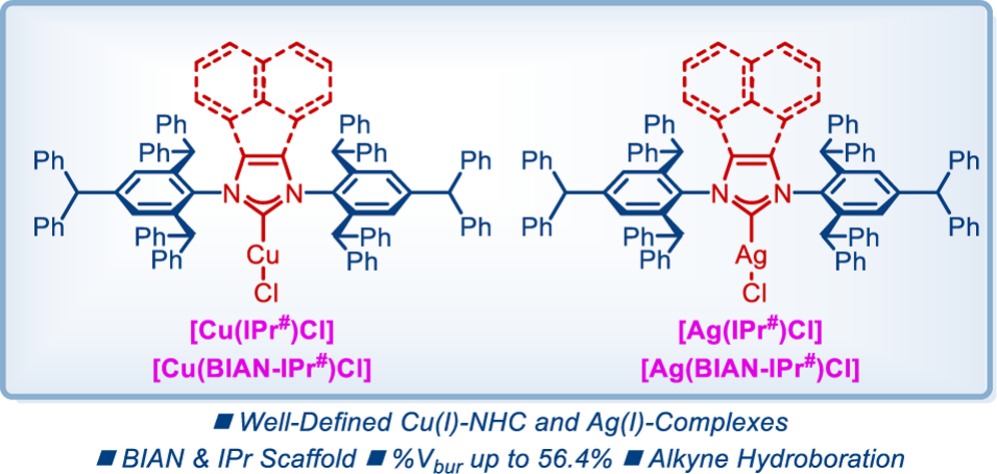

Metal–N-heterocyclic carbene (M–NHC) complexes are well-known as an important class of organometallic compounds widely used in transition-metal catalysis. Taking into account that the steric hindrance around the metal center is one of the major effects in M–NHC catalysis, the development of new, sterically hindered M–NHC complexes is an ongoing interest in this field of research. Herein, we report the synthesis and characterization of exceedingly sterically hindered, well-defined, air- and moisture-stable Cu(I) and Ag(I) complexes, [Cu(NHC)Cl] and [Ag(NHC)Cl], in the recently discovered IPr^#^ family of ligands that hinge upon modular peralkylation of anilines. The complexes in both the BIAN and IPr families of ligands are reported. X-ray crystallographic analyses and computational studies were conducted to determine steric effects, Frontier molecular orbitals, and bond orders. The complexes were evaluated in the model hydroboration of the alkynes. We identified [Cu(BIAN–IPr^#^)Cl] and [Ag(BIAN–IPr^#^)Cl] as highly reactive catalysts with the reactivity outperforming the classical IPr and IPr*. Considering the attractive features of well-defined Cu(I)–NHC and Ag(I)–NHC complexes, this class of sterically bulky yet wingtip-flexible complexes will be of interest for catalytic processes in various areas of organic synthesis and catalysis.

## 1 Introduction

N-Heterocyclic carbenes (NHCs) have emerged as a powerful class of ligands for transition-metal catalysis in organic synthesis and organometallic chemistry.^1−3^ This important class of ancillary ligands exhibits unique steric, electronic, and structural properties that favor the kinetic stability of metal–NHC complexes and protect the catalytic species.^4−6^ Consequently, NHCs have become universal ligands that are capable of coordinating various metals at different oxidation states.^7^ In this context, the utilization of high steric effects of NHC ligands has been a major driving force in the study of the structural properties and catalytic reactivity.^1−7^

Metal–NHC complexes of group 11 are considered to be an important class of catalysts for π-activation reactions. In particular, [Au(NHC)X],^8^ [Cu(NHC)X],^9^ and [Ag(NHC)X] complexes^10^ are of major significance in alkyne functionalization reactions;^11,12^ however, their reactivity is by no means limited to promoting synthetically valuable π-functionalizations.^8−10^

On the other hand, the steric and electronic properties of NHC ligands play a vital role in stabilizing metal centers. Consequently, studies have been performed to investigate the effect of steric and electronic properties of NHC ligands on the stability and catalytic activity of the copper and silver complexes.^9,10^ Notably, the vast majority of studies have focused on classical imidazole-2-ylidene ligands and selected abnormal NHC ligands. The effects of exceedingly bulky substitution and the steric flexibility of the NHC ligands remain little explored.

**Figure 1:**
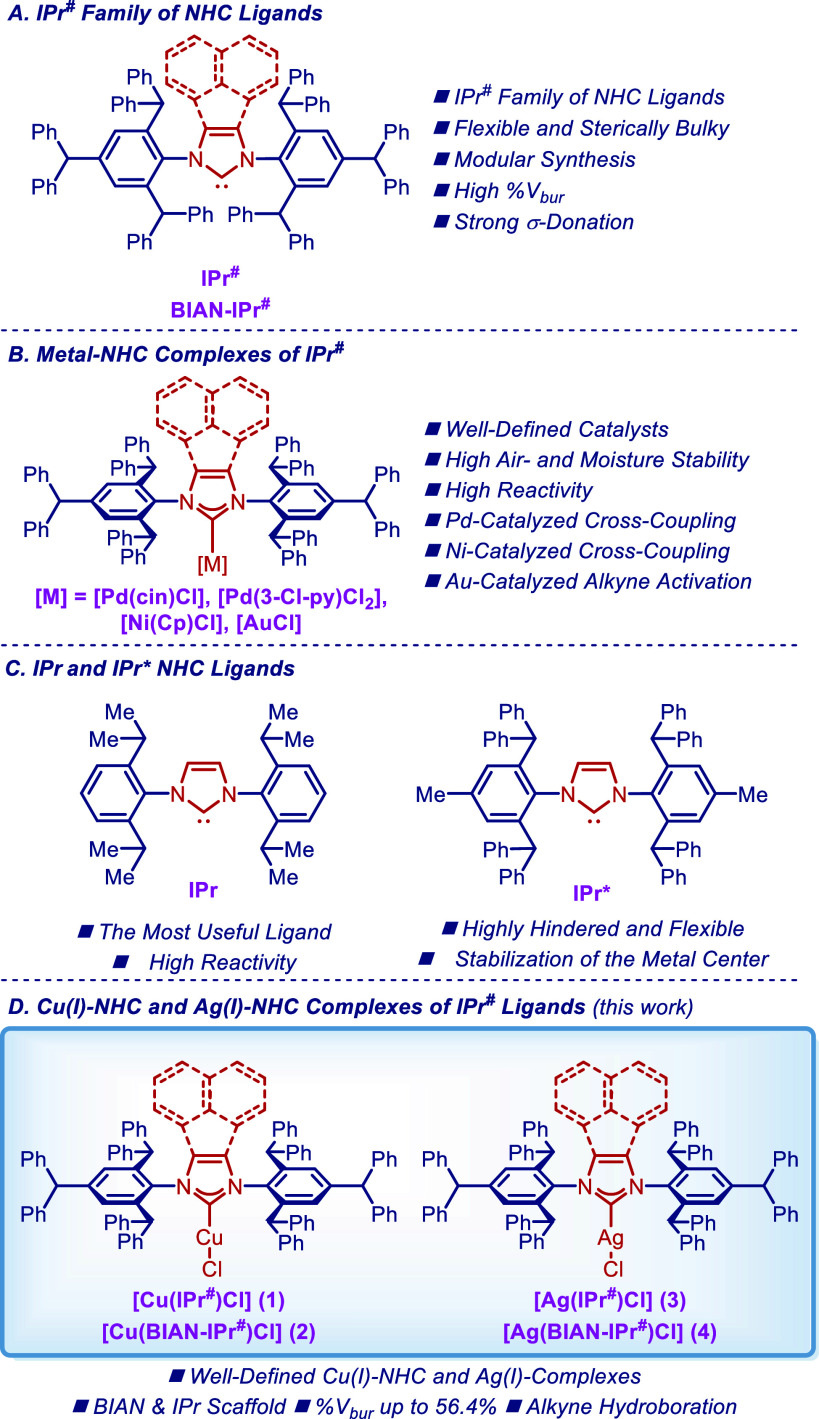
(A–D) IPr-based NHC ligands and their metal–NHC complexes.

Recently, our laboratory introduced IPr^#^ ligands, which represent a new class of Arduengo-type NHCs that hinge upon a modular peralkylation of aniline (Figure 1A).^13^ These ligands have been commercialized in collaboration with Millipore Sigma to provide broad access to academic and industrial researchers for reaction screening and optimization using sterically bulky NHC ligands.^14^ To date, several classes of M–IPr^#^ have been prepared, including Pd, Ni, and Au (Figure 1B). Most importantly, the described complexes exhibit high and general reactivity in Pd- and Ni-catalyzed cross-coupling reactions by activation of C–X, C–N, C–O, C–S, and C–H bonds.^15,16^ Furthermore, Au(I)–NHC catalysts showed high reactivity in alkyne hydration and hydrocarboxylation reactions with the activity higher than IPr (1,3-bis(2,6-diisopropylphenyl)imidazole-2-ylidene) and IPr* (1,3-bis(2,6-bis(diphenylmethyl)-4-methylphenyl)imidazo-2-ylidene) congeners (Figure 1C).^17^ Recently, Ag(I) and Cu(I) complexes bearing the IPr^#^ ligand were reported.^18^ However, the methods described led to products in moderate yields. For example, the use of Cu_2_O in analogy to Ag_2_O gave the complex [Cu(IPr^#^)Cl] in only 37%.^18b^

Herein, as a part of our program in ligand design and catalysis,^16^ we report the efficient synthesis and characterization of exceedingly hindered, air- and moisture-stable, well-defined [Cu(NHC)Cl] and [Ag(NHC)Cl] complexes based on the IPr# scaffold. The synthesis of complexes in both BIAN and IPr series is reported (Figure 1D). X-ray crystallographic analysis and computational studies were conducted to determine steric effects, Frontier molecular orbitals, and bond orders. The complexes were evaluated in the model hydroboration of alkynes. We identified [Cu(BIAN–IPr^#^)Cl] and [Ag(BIAN–IPr^#^)Cl] as highly reactive catalysts, with the reactivity outperforming the classical IPr and IPr*. Considering the attractive features of well-defined Cu(I)–NHC and Ag(I)–NHC complexes, this class of sterically bulky yet wingtip-flexible complexes will be of interest for catalytic processes in various areas of organic synthesis and catalysis.

## 2 Results and Discussion

### 2.1 Research Design

For this study, we selected well-defined [Cu(IPr^#^)Cl] (**1**), [Cu(BIAN–IPr^#^)Cl] (**2**), [Ag(IPr^#^)Cl] (**3**), and [Ag(BIAN–IPr^#^)Cl] (**4**) complexes from the IPr^#^ family of NHC ligands (Figure 1D). Classical imidazole-2-ylidene-based NHC complexes [Cu(IPr*)Cl] (**5**), [Cu(IPr)Cl] (**6**), [Ag(IPr*)Cl] (**7**), and [Ag(IPr)Cl] (**8**) were selected for comparison of structural, electronic, and steric properties.

### 2.2 Complex Synthesis

**Scheme 1:**
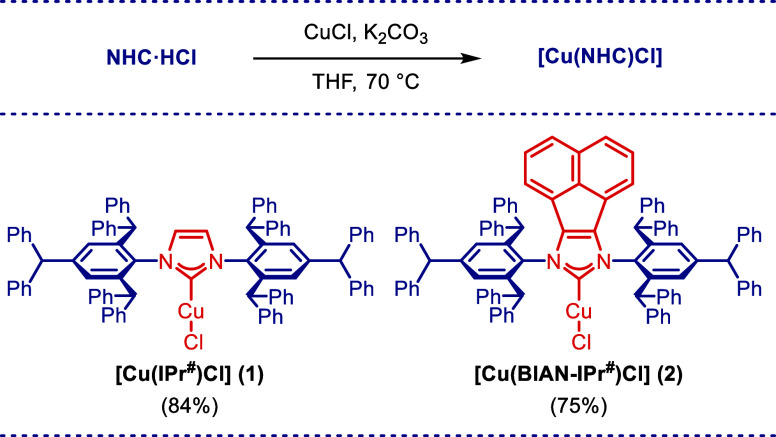
Synthesis of Cu(I)–NHC Complexes Conditions: NHC·HCl (1.0 equiv), CuCl (1.5 equiv), K_2_CO_3_ (3.0 equiv), THF (0.05 M), 70 °C.

**Scheme 2:**
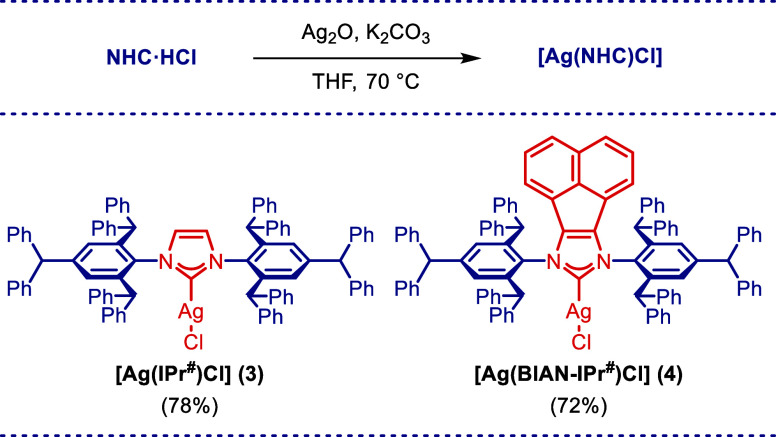
Synthesis of Ag(I)–NHC Complexes Conditions: NHC·HCl (1.0 equiv), Ag_2_O (1.5 equiv), K_2_CO_3_ (3.0 equiv), THF (0.05 M), 70 °C.

We initiated our studies by developing an efficient synthesis of well-defined Cu(I) and Ag(I) complexes of the IPr^#^ family of ligands. The corresponding complexes [Cu(IPr^#^)Cl] (**1**) and [Cu(BIAN–IPr^#^)Cl] (**2**) were synthesized by the reaction of NHC·HCl salts with CuCl in the presence of K_2_CO_3_ in THF at 70 °C (Scheme 1). Silver complexes [Ag(IPr^#^)Cl] (**3**) and [Ag(BIAN–IPr^#^)Cl] (**4**) were obtained by using the same procedure in the presence of Ag_2_O (Scheme 2). These complexes were isolated in 72–84% yield by trituration with hexane. All complexes were found to be stable to air and moisture. The method affords the desired products **1–4** with high efficiency compared to other protocols.^18^

### 2.3 Crystallographic Studies

**Figure 2:**
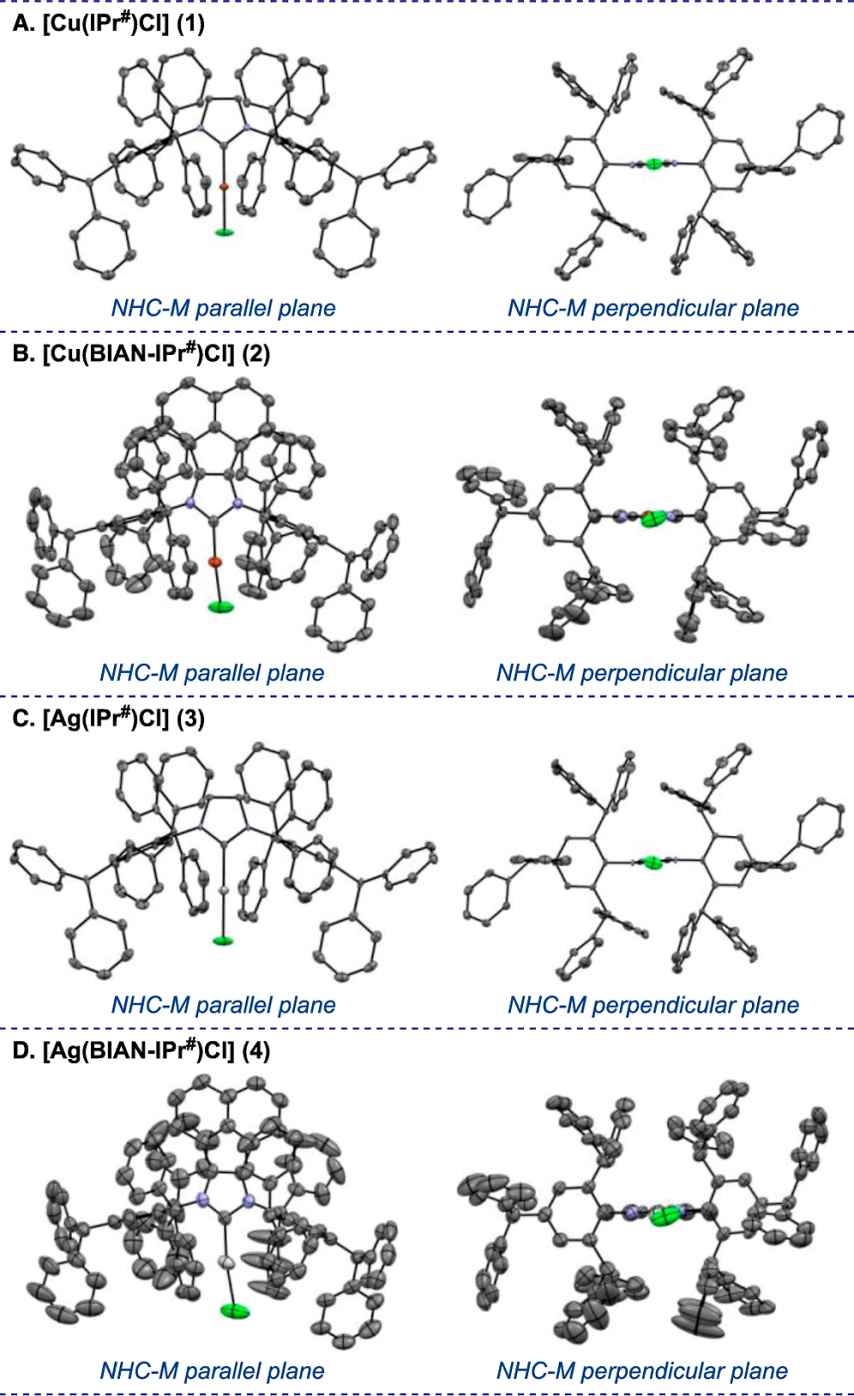
X-ray crystal structure of [M(NHC)Cl] complexes (**1–4**) (50% ellipsoids). Selected bond lengths [Å], bond angles [°], and dihedral angles [°]: (A) [Cu(IPr^#^)Cl] (**1**): Cu1–Cl1, 2.109(9); Cu1–C1, 1.885(3); C1–N1, 1.355(2); N1–C3, 1.443(2); Cl1–Cu1–C1, 180.00; Cu1–C1–N1, 127.91(11); C1–N1–C3, 122.48(16); Cu1–C1–N1–C3, 4.40; C3–N1–N1–C3, 21.98. (B) [Cu(BIAN–IPr^#^)Cl] (**2**): Cu1–Cl1, 2.0898(13); Cu1–C1, 1.869(3); C1–N1, 1.375(4); C1–N2, 1.368(4); N1–C14, 1.434(4), N2–C59, 1.448(4); Cl1–Cu1–C1, 174.57(11); Cu1–C1–N1, 124.2(2); Cu1–C1–N2, 130.7(2); C1–N1–C14, 122.4(3); C1–N2–C59, 124.4(3); Cu1–C1–N1–C14, 2.44; Cu1–C1–N2–C59, 4.21; C14–N1–N2–C59, 4.79. (C) [Ag(IPr^#^)Cl] (**3**): Ag1–Cl1, 2.2983(17); Ag1–C1, 2.071(5); C1–N1, 1.348(4); N1–C4, 1.448(4); Cl1–Ag1–C1, 180.0; Ag1–C1–N1, 127.3(2); C1–N1–C4, 122.9(3); Ag1–C1–N1–C4, 4.82; C4–N1–N1–C4, 23.64. (D) [Ag(BIAN–IPr^#^)Cl] (**4**): Ag1–Cl1, 2.308(2); Ag1–C1, 2.078(5); C1–N1, 1.377(6); C1–N2, 1.343(6); N1–C14, 1.435(6), N2–C59, 1.437(6); Cl1–Ag1–C1, 172.31(14); Ag1–C1–N1, 122.8(3); Ag1–C1–N2, 130.9(4); C1–N1–C14, 122.2(4); C1–N2–C59, 124.6(4); Ag1–C1–N1–C14, 4.95; Ag1–C1–N2–C59, 2.80; C14–N1–N2–C59, 5.09. CCDC 2373450 (**1**). CCDC 2373451 (**2**). CCDC 2373455 (**3**). CCDC 2373458 (**4**).

**Figure 3:**
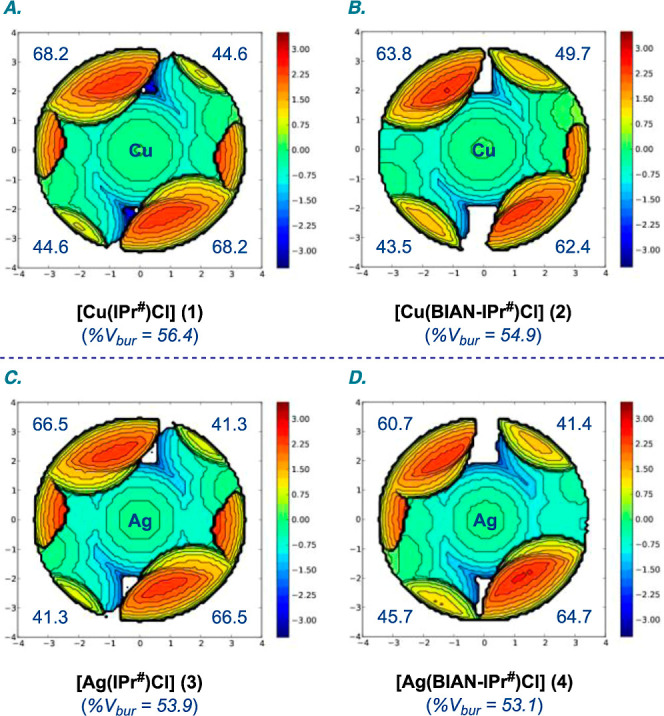
(A–D) Topographical steric maps of complexes [M(NHC)Cl] **1–4** showing %*V*_bur_ per quadrant (*r* = 3.5 Å).

Extensive studies by Nolan, Cavallo, and co-workers showed that the combination of percent buried volume (%*V*_bur_) and steric maps of linear metal–NHC complexes provides the most accurate model to determine the overall steric effect of NHC ligands.^5^ Thus, the synthesized complexes **1–4** were fully characterized by single-crystal X-ray analysis (Figures 2 and 3 and Table 1, see Supporting Information for additional details). The crystals were grown by the slow solvent diffusion method using dichloromethane and *n*-hexane. The monoclinic or triclinic crystal packing unit cell consists of one molecule for each [Cu(IPr^#^)Cl] (**1**), [Cu(BIAN–IPr^#^)Cl] (**2**), [Ag(IPr^#^)Cl] (**3**), and [Ag(BIAN–IPr^#^)Cl] (**4**) complex.

**Table 1:** Selected Angles and Bond Lengths of [Cu(NHC)Cl] and [Ag(NHC)Cl] Complexes

structure	C–M–Cl (°) M = Cu, Ag	M–C (Å) M = Cu, Ag	M–Cl (Å) M = Cu, Ag
[Cu(IPr^#^)Cl]	180.00	1.885(3)	2.109(9)
[Cu(BIAN–IPr^#^)Cl]	174.57(11)	1.869(3)	2.0898(13)
[Cu(IPr*)Cl]^a^	176.21(9)	1.867(3)	2.0944(9)
[Cu(IPr)Cl]^b^	180.00	1.953	2.089
[Ag(IPr^#^)Cl]	180.00	2.071(5)	2.2983(17)
[Ag(BIAN–IPr^#^)Cl]	172.31(14)	2.078(5)	2.308(2)
[Ag(IPr*)Cl]^c^	174.29(7)	2.081(2)	2.3189(9)
[Ag(IPr)Cl]^d^	175.20(2)	2.056(6)	2.316(17)

a Ref (8k).

b Ref (19).

c Ref (20).

d Ref (21).

Similar to other Cu(I)–NHC complexes, [Cu(IPr^#^)Cl] (**1**) and [Cu(BIAN–IPr^#^)Cl] (**2**) have a bi-coordinated linear geometry (Figure 2A,B). The bond angles associated with the metal centers of [Cu(IPr^#^)Cl] (**1**) (C–Cu–Cl, 180.00°) and [Cu(BIAN–IPr^#^)Cl] (**2**) (C–Cu–Cl, 174.57°) are consistent with the linear geometry and in the range of reported Cu(I)–NHC complexes, such as [Cu(IPr*)Cl] (**5**) (C–Cu–Cl, 176.21°) and [Cu(IPr)Cl] (**6**) (C–Cu–Cl, 180.00°) (Table 1).^8k,19^

The metal–ligand and metal–halogen bond lengths of [Cu(IPr^#^)Cl] (**1**) (Cu–C, 1.885 Å; Cu–Cl, 2.109 Å) and [Cu(BIAN–IPr^#^)Cl] (**2**) (Cu–C, 1.869 Å; Cu–Cl, 2.090 Å) can be compared with the reported [Cu(NHC)Cl] complexes {[Cu(IPr*)Cl] (**5**), Cu–C, 1.867 Å; Cu–Cl, 2.094 Å and [Cu(IPr)Cl] (**6**), Cu–C, 1.953 Å; Cu–Cl, 2.089 Å}.^8k,19^

Furthermore, crystallographic analysis of [Ag(IPr^#^)Cl] (**3**) and [Ag(BIAN–IPr^#^)Cl] (**4**) showed the characteristic linear geometry (Figure 2C,D). The distance of metal–carbene and metal–halide bonds for [Ag(IPr^#^)Cl] (**3**) (Ag–C, 2.071 Å; Ag–Cl, 2.298 Å) and [Ag(BIAN–IPr^#^)Cl] (**4**) (Ag–C, 2.078 Å; Ag–Cl, 2.308 Å) can be compared with the reported [Ag(NHC)Cl] complexes {[Ag(IPr*)Cl] (**7**), Ag–C, 2.082 Å; Ag–Cl, 2.319 Å and [Ag(IPr)Cl] (**8**), Ag–C, 2.056 Å; Ag–Cl, 2.313 Å} (Table 1).^20,21^ The bond angles of [Ag(IPr^#^)Cl] (**3**) (C–Ag–Cl, 180.00°) and [Ag(BIAN–IPr^#^)Cl] (**4**) (C–Ag–Cl, 172.31°) indicate the linear geometry, which are in a similar range of the reported [Ag(IPr*)Cl] (**7**) (C–Ag–Cl, 174.29°) and [Ag(IPr)Cl] (**8**) (C–Ag–Cl, 175.20°) complexes.

To further evaluate the steric impact of IPr^#^ and BIAN–IPr^#^ ligands, the percent buried volume (%*V*_bur_) with quadrant distribution of the ligand on the metal coordination sphere was determined using the method developed by Cavallo (Figure 3 and Table 2).^5b^ The %*V*_bur_ of [Cu(IPr^#^)Cl] (**1**) is 56.4%, and that of [Cu(BIAN–IPr^#^)Cl] (**2**) is 54.9%. These values can be compared with 52.1% and 48.5% determined for [Cu(IPr*)Cl] (**5**) and [Cu(IPr)Cl] (**6**), respectively. For Ag(I)–NHC complexes, the %*V*_bur_ of [Ag(IPr^#^)Cl] (**3**) is 53.9%, and that of [Ag(BIAN–IPr^#^)Cl] (**4**) is 53.1%; these values can be compared with the reported complexes [Ag(IPr*)Cl] (**7**) %*V*_bur_ 53.5% and [Ag(IPr)Cl] (**8**) %*V*_bur_ 43.8%. A detailed comparison of the steric properties, including quadrant distribution, is shown in Table 2. Note that in this case, %*V*_bur_ refers to the comparison of isoelectronic and isosteric ligands.^5^

**Table 2:** Comparison of %*V*_bur_ of Copper and Silver Complexes

structure	%*V*_bur_				
	SW	NW	NE	SE	total
[Cu(IPr^#^)Cl]	44.6	68.2	44.6	68.2	56.4
[Cu(BIAN–IPr^#^)Cl]	43.5	63.8	49.7	62.4	54.9
[Cu(IPr*)Cl]^a^	63.1	54.9	42.5	48.1	52.1
[Cu(IPr)Cl]^b^	47.5	49.5	47.5	49.5	48.5
[Ag(IPr^#^)Cl]	41.3	66.5	41.3	66.5	53.9
[Ag(BIAN–IPr^#^)Cl]	45.7	60.7	41.4	64.7	53.1
[Ag(IPr*)Cl]^c^	41.0	52.6	52.2	68.3	53.5
[Ag(IPr)Cl]^d^	52.4	33.7	38.1	51.1	43.8

a Ref (8k).

b Ref (19).

c Ref (20).

d Ref (21).

### 2.4 Computational Studies

To gain insights into the steric and electronic properties of this class of M–NHCs, Cu(I) complexes [Cu(IPr^#^)Cl] (**1**) and [Cu(BIAN–IPr^#^)Cl] (**2**) were further evaluated at the B3LYP 6-311++g(d,p) level. [Cu(IPr*)Cl] (**5**) and [Cu(BIAN–IPr*)Cl] (**9**) were selected for comparison.

**Figure 4:**
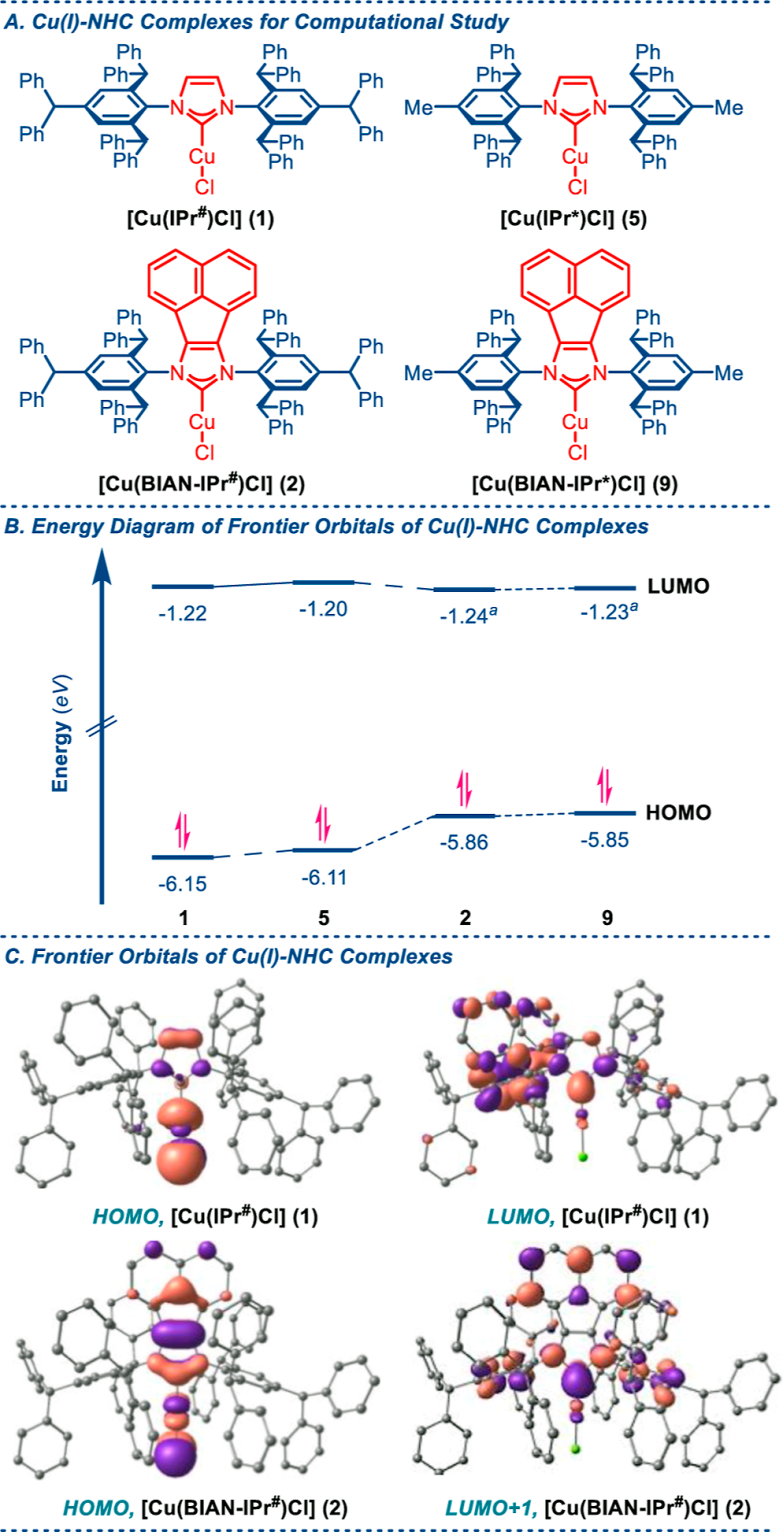
(A–C) Structures, energy diagram, and frontier orbitals of Cu(I)–NHCs.

First, we evaluated the electronic properties (Figure 4). The HOMOs are located within the Cu–Cl bond, and the LUMOs are located within the Cu–C_carbene_ bond. The HOMO of [Cu(IPr^#^)Cl] (**1**) (−6.15 eV) can be compared with the HOMO of [Cu(IPr*)Cl] (**5**) (−6.11 eV). The HOMO of [Cu(BIAN–IPr^#^)Cl] (**2**) (−5.86 eV) is higher than that of **1** and **5**, and this value is in a range similar to the HOMO of [Cu(BIAN–IPr*)Cl] (**9**) (−5.85 eV). The LUMO values of [Cu(IPr^#^)Cl] (**1**) (−1.22 eV) and [Cu(BIAN–IPr^#^)Cl] (**2**) (−1.24 eV) can be compared with the LUMO values of [Cu(IPr*)Cl] (**5**) (−1.20 eV) and [Cu(BIAN-IPr*)Cl] (**9**) (−1.23 eV). Overall, this analysis indicates that the Cu–C_carbene_ bond of [Cu(IPr^#^)Cl] (**1**), [Cu(BIAN–IPr^#^)Cl] (**2**), [Cu(IPr*)Cl] (**5**), and [Cu(BIAN–IPr*)Cl] (**9**) has similar electronic stability. However, the HOMO of [Cu(BIAN–IPr^#^)Cl] (**2**) and [Cu(BIAN–IPr*)Cl] (**9**) is relatively higher than that of [Cu(IPr^#^)Cl] (**1**) and [Cu(IPr*)Cl] (**5**). These observations are important for π-functionalization reactions.

The computed metal–ligand and metal–halogen bond lengths of [Cu(IPr^#^)Cl] (**1**) (Cu–C, 1.908 Å; Cu–Cl, 2.138 Å) and [Cu(BIAN–IPr^#^)Cl] (**2**) (Cu–C, 1.908 Å; Cu–Cl, 2.144 Å) are slightly longer than those observed from X-ray analysis (Table 3). These values can be compared with the [Cu(NHC)Cl] complexes {[Cu(IPr*)Cl] (**5**), Cu–C, 1.907 Å; Cu–Cl, 2.137 Å; and [Cu(BIAN–IPr*)Cl] (9), Cu–C, 1.908 Å; Cu–Cl, 2.145 Å}.

**Table 3:** Computed Angles and Bond Lengths of [Cu(NHC)Cl] Complexes

structure	C–Cu–Cl (°)	Cu–C (Å)	Cu–Cl (Å)
[Cu(IPr^#^)Cl]	178.436	1.908	2.138
[Cu(IPr*)Cl]	178.509	1.907	2.137
[Cu(BIAN–IPr^#^)Cl]	180.000	1.908	2.144
[Cu(BIAN–IPr*)Cl]	179.997	1.908	2.145

To gain further insights into the relative strength of the metal–ligand bond, the Wiberg bond order analysis was performed (for more details, see Supporting Information, Table S5), which indicated that the Cu–C_carbene_ bonds in [Cu(IPr^#^)Cl] (**1**), [Cu(BIAN–IPr^#^)Cl] (**2**), [Cu(IPr*)Cl] (**5**), and [Cu(BIAN–IPr*)Cl] (**9**) have similar bond orders.

**Figure 5:**
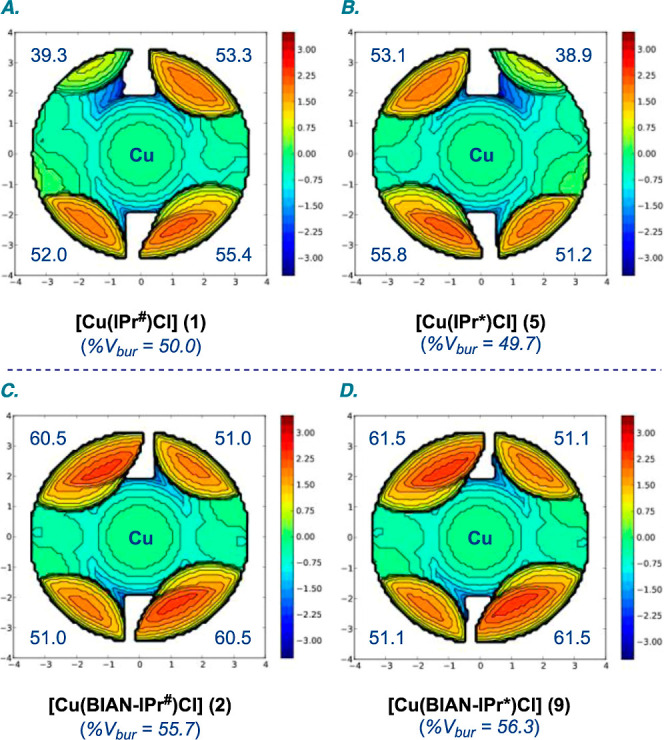
(A–D) Topographical steric maps of [Cu(NHC)Cl] showing %*V*_bur_ per quadrant [B3LYP 6-311++g(d,p)].

To better understand the steric impact and eliminate effects resulting from crystal packing, the percent buried volume (%*V*_bur_) was calculated from the optimized structures (Figure 5). The %*V*_bur_ of [Cu(IPr^#^)Cl] (**1**) is 50.0% (SW, 52.0%; NW, 39.3%; NE, 53.3%; SE, 55.4%), which can be compared with [Cu(IPr*)Cl] (**5**) (%*V*_bur_, 49.7%; SW, 55.8%; NW, 53.1%; NE, 38.9%; SE, 51.2%). The steric impact of [Cu(BIAN–IPr^#^)Cl] (**2**) (%*V*_bur_, 55.7%; SW, 51.0%; NW, 60.5%; NE, 51.0%; SE, 60.5%) can be compared with [Cu(BIAN–IPr*)Cl] (**9**) (%*V*_bur_, 56.3%; SW, 51.1%; NW, 61.5%; NE, 51.1%; SE, 61.5%). Overall, the higher steric impact of BIAN–NHCs is consistent with more precise control of the catalytic pocket and an increased protection of the metal center in this scaffold. However, the most reliable comparison is made through a combination of X-ray and DFT studies.

### 2.5 Catalyst Evaluation

Next, we evaluated the catalytic reactivity of [Cu(IPr^#^)Cl] (**1**) and [Cu(BIAN–IPr^#^)Cl] (**2**) as well as [Ag(IPr^#^)Cl] (**3**) and [Ag(BIAN–IPr^#^)Cl] (**4**) in the model hydroboration of alkynes and compared their reactivity with classical [Cu(IPr*)Cl] (**5**) and [Cu(IPr)Cl] (**6**), [Ag(IPr*)Cl] (**7**), and [Ag(IPr)Cl] (**8**) (Tables 4 and 5).

**Table 4:**
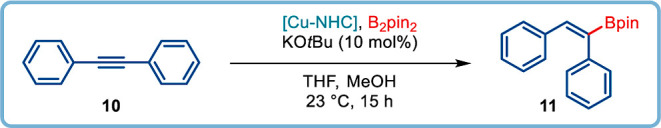
Hydroboration of Alkynes Using [Cu(NHC)Cl] Complexes^a^

entry	[Cu(I)–NHC] catalyst	yield (%)
1	[Cu(IPr^#^)Cl]	87
2	[Cu(BIAN–IPr^#^)Cl]	97
3	[Cu(IPr*)Cl]	82
4	[Cu(IPr)Cl]	70

a Conditions: diphenylacetylene (1.0 equiv), B_2_pin_2_ (1.2 equiv), [Cu(NHC)Cl] (0.5 mol %), KO*t*Bu (10.0 mol %), MeOH/THF, 23 °C, 15 h.

**Table 5:**
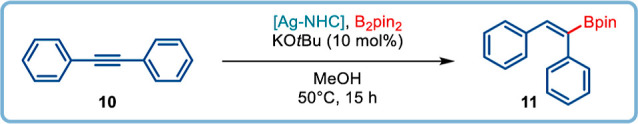
Hydroboration of Alkynes Using [Ag(NHC)Cl] Complexes^a^

entry	[Ag(I)–NHC] catalyst	yield (%)
1	[Ag(IPr^#^)Cl]	17
2	[Ag(BIAN–IPr^#^)Cl]	57
3	[Ag(IPr*)Cl]	36
4	[Ag(IPr)Cl]	10

a Conditions: diphenylacetylene (1.0 equiv), B_2_pin_2_ (1.2 equiv), [Ag(NHC)Cl] (2.0 mol %), KO*t*Bu (10.0 mol %), MeOH, 50 °C, 15 h.

Cu(I)–NHC-catalyzed and Ag(I)–NHC-catalyzed hydroboration of alkynes was selected as a model reaction owing to the importance of this class of reactions to access valuable functionalized products and intermediates for chemical and pharmaceutical synthesis. The results showed that the catalyst [Cu(BIAN–IPr^#^)Cl] (**2**) outperformed other catalysts in the Cu(I)–NHC-catalyzed hydroboration, while [Ag(BIAN–IPr^#^)Cl] (**4**) was also the most reactive in the series.

## 3 Conclusions

In summary, we have reported the synthesis and characterization of exceedingly sterically hindered, air- and moisture-stable, well-defined [Cu(IPr^#^)Cl], [Cu(BIAN–IPr^#^)Cl], [Ag(IPr^#^)Cl], and [Ag(BIAN–IPr^#^)Cl] complexes. All complexes were characterized by single-crystal X-ray analysis, which confirmed the linear geometry and steric effects of the NHC ligand on the metal center. The structural and steric properties were compared with those of classical [Cu(IPr*)Cl], [Cu(IPr)Cl], [Ag(IPr*)Cl], and [Ag(IPr)Cl] complexes. The electronic properties were evaluated by a computational analysis. The reactivity was evaluated in the model hydroboration of alkynes, where [Cu(BIAN–IPr^#^)Cl] and [Ag(BIAN–IPr^#^)Cl] outperformed other complexes. Considering the high interest in well-defined Cu(I)–NHCs and Ag(I)–NHCs, we anticipate that this class of sterically bulky complexes will find applications in catalysis of various organic transformations.

## 4 Experimental Section

### 4.1 General Information

All starting materials reported in the manuscript have been previously described in the literature and prepared by the method reported previously unless stated otherwise. All experiments were performed using standard Schlenk techniques under nitrogen or argon unless stated otherwise. All solvents were purchased at the highest commercial grade and used as received or after purification by passing through activated alumina columns or distillation from sodium/benzophenone under nitrogen. All solvents were deoxygenated prior to use. All other chemicals were purchased at the highest commercial grade and used as received. Reaction glassware was oven-dried at 140 °C for at least 24 h or flame-dried prior to use, allowed to cool under vacuum, and purged with argon (three cycles). All products were identified using ^1^H NMR analysis and comparison with authentic samples. GC or GC/MS analysis was used for volatile products. All yields refer to yields determined by ^1^H NMR and/or GC or GC/MS using an internal standard (optimization) and isolated yields (preparative runs) unless stated otherwise. ^1^H and ^13^C NMR spectra were recorded in CDCl_3_ on Bruker spectrometers at 500 (^1^H NMR) and 125 MHz (^13^C NMR). All shifts are reported in parts per million (ppm) relative to the residual CHCl_3_ peak (7.26 and 77.16 ppm, ^1^H NMR and ^13^C NMR, respectively). All coupling constants (*J*) are reported in hertz (Hz). Abbreviations are s, singlet; d, doublet; t, triplet; q, quartet; and brs, broad singlet. GC–MS chromatography was performed using an Agilent HP6890 GC system and an Agilent 5973A inert XL EI/CI MSD using helium as the carrier gas at a flow rate of 1 mL/min and an initial oven temperature of 50 °C. The injector temperature was 250 °C. The detector temperature was 250 °C. For runs with the initial oven temperature of 50 °C, the temperature was increased with a 10 °C/min ramp after 50 °C hold for 3 min to a final temperature of 220 °C and then held at 220 °C for 15 min (split less mode of injection; total run time of 22.0 min). High-resolution mass spectra (HRMS) were measured on a 7T Bruker Daltonics FT-MS instrument. All flash chromatography was performed using silica gel (60 Å, 300 mesh). TLC analysis was carried out on glass plates coated with silica gel 60 F254, 0.2 mm thickness. The plates were visualized by using a 254 nm UV lamp or aqueous potassium permanganate. ^1^H NMR and ^13^C NMR data are given for all compounds in the Supporting Information experimental section for characterization purposes. ^1^H NMR, ^13^C NMR, and HRMS data are given for all new compounds.

### 4.2 Experimental Procedures and Characterization Data

**IPr**^**#**^**·HCl** and **BIAN–IPr#·HCl** are commercially available and previously reported in the literature.^13^ Compound **11**(16a) has been previously reported in the literature, and spectroscopic properties matched literature data.

#### 4.2.1 General Procedure for the Synthesis of [Cu(NHC)Cl] Complexes

An oven-dried pressure tube equipped with a stir bar was charged with NHC·HCl salt (0.1 mmol, 1.0 equiv), CuCl (0.15 mmol, 148 mg, 1.5 equiv), and K_2_CO_3_ (0.3 mmol, 41 mg, 3.0 equiv), placed under a positive pressure of argon, and subjected to three evacuation/backfilling cycles under high vacuum. THF (0.05 M) was added, and the reaction mixture was stirred at 70 °C for 15 h. After the indicated time, the reaction mixture was diluted with CH_2_Cl_2_ (10 mL) and filtered. The solution was collected and concentrated. The title products were obtained by trituration from hexanes.

#### 4.2.2 Characterization Data for [Cu(NHC)Cl] Complexes

[Cu(IPr^#^)Cl] (**1**). White solid. Yield 84% (111 mg). ^1^H NMR (500 MHz, CDCl_3_) δ: 7.19 (t, *J* = 7.1 Hz, 8H), 7.14 (t, *J* = 7.1 Hz, 16H), 7.10–7.03 (m, 12H), 6.94 (d, *J* = 6.7 Hz, 8H), 6.89 (dd, *J* = 7.4, 2.2 Hz, 8H), 6.83–6.72 (m, 12H), 5.88 (s, 2H), 5.39 (s, 2H), 5.18 (s, 4H). ^13^C NMR (126 MHz, CDCl_3_) δ 180.50, 145.79, 143.14, 143.07, 142.18, 141.04, 134.74, 130.90, 129.45, 129.39, 129.36, 128.64, 128.49, 128.43, 126.72, 126.63, 126.45, 123.28, 56.29, 51.44. HRMS (ESI) *m*/*z*: [M + Na]^+^ calcd for C_93_H_72_CuClN_2_Na, 1337.4578; found, 1337.4632.

[Cu(BIAN–IPr^#^)]Cl] (**2**). Yellow solid. Yield 75% (108 mg). ^1^H NMR (500 MHz, CDCl_3_) δ: 7.54 (d, *J* = 8.3 Hz, 2H), 7.24 (t, *J* = 7.4 Hz, 8H), 7.17 (t, *J* = 7.3 Hz, 4H), 7.04–6.95 (m, 18H), 6.96–6.90 (m, 8H), 6.89–6.83 (m, 8H), 6.75 (t, *J* = 7.2 Hz, 4H), 6.70 (t, *J* = 7.3 Hz, 8H), 6.62 (d, *J* = 6.9 Hz, 8H), 6.11 (d, *J* = 6.9 Hz, 2H), 5.48 (s, 2H), 5.37 (s, 4H). ^13^C NMR (126 MHz, CDCl_3_) δ: 185.80, 145.77, 143.25, 142.09, 141.79, 141.74, 138.99, 133.96, 130.99, 129.72, 129.66, 129.43, 129.31, 128.48, 128.44, 128.03, 127.32, 126.50, 126.35, 124.16, 121.50, 56.37, 51.59. HRMS (ESI) *m*/*z*: [M + Na]^+^ calcd for C_103_H_76_CuClN_2_Na, 1461.4891; found, 1461.4903.

#### 4.2.3 General Procedure for the Synthesis of [Ag(NHC)Cl] Complexes

An oven-dried pressure tube equipped with a stir bar was charged with NHC·HCl salt (0.1 mmol, 1.0 equiv), Ag_2_O (0.15 mmol, 35 mg, 1.5 equiv), and K_2_CO_3_ (0.3 mmol, 41 mg, 3.0 equiv), placed under a positive pressure of argon, and subjected to three evacuation/backfilling cycles under high vacuum. THF (0.05 M) was added, and the reaction mixture was stirred at 70 °C for 15 h. After the indicated time, the reaction mixture was diluted with CH_2_Cl_2_ (10 mL) and filtered. The solution was collected and concentrated. The title products were obtained by trituration from hexanes.

#### 4.2.4 Characterization Data for [Ag(NHC)Cl] Complexes

[Ag(IPr^#^)Cl] (**3**). White solid. Yield 78% (106 mg). ^1^H NMR (500 MHz, CDCl_3_) δ: 7.21–7.18 (m, 8H), 7.17–7.13 (m, 16H), 7.09–7.08 (m, 12H), 6.95 (d, *J* = 7.1 Hz, 8H), 6.82–6.78 (m, 20H), 6.03 (d, *J* = 1.7 Hz, 2H), 5.40 (s, 2H), 5.11 (s, 4H). ^13^C NMR (126 MHz, CDCl_3_) δ: 184.62 (d, *J*^109^Ag-^13^C = 268.6 Hz, *J*^107^Ag-^13^C = 232.7 Hz), 145.98, 143.12, 143.04, 141.93, 140.94, 134.86, 130.98, 129.37, 129.35, 129.32, 128.82, 128.54, 128.46, 126.81, 126.73, 126.48, 123.73, 123.67, 56.30, 51.39. HRMS (ESI) *m*/*z*: [M – Cl]^+^ calcd for C_93_H_72_AgN_2_, 1323.4746; found, 1323.4749.

[Ag(BIAN–IPr^#^)Cl] (**4**). Yellow solid. Yield 72% (107 mg). ^1^H NMR (500 MHz, CDCl_3_) δ: 7.59 (d, *J* = 8.3 Hz, 2H), 7.25 (t, *J* = 7.3 Hz, 8H), 7.18 (t, *J* = 7.3 Hz, 4H), 7.06 (t, *J* = 7.6 Hz, 2H), 7.01 (d, *J* = 7.5 Hz, 8H), 6.99–6.92 (m, 12H), 6.92 (s, 4H), 6.85–6.66 (m, 20H), 6.63 (d, *J* = 7.3 Hz, 8H), 6.17 (d, *J* = 6.9 Hz, 2H), 5.49 (s, 2H), 5.31 (s, 4H). ^13^C NMR (126 MHz, CDCl_3_) δ 189.66 (d, *J*^109^Ag-^13^C = 271.5 Hz), 145.99, 143.13, 141.88, 141.83, 141.63, 139.47, 139.41, 134.03, 131.04, 129.60, 129.41, 129.16, 128.64, 128.51, 128.08, 127.55, 126.62, 126.53, 126.41, 124.03, 121.56, 56.35, 51.53. HRMS (ESI) *m*/*z*: [M + Na]^+^ calcd for C_103_H_76_AgClN_2_Na, 1505.4646; found, 1505.4660.

#### 4.2.5 General Procedure for [Cu–NHC]-Catalyzed Hydroboration of Alkynes

An oven-dried vial equipped with a stir bar was charged with diphenylacetylene (0.5 mmol, 89 mg, 1.0 equiv), bis(pinacolato)diboron (0.6 mmol, 152 mg, 1.2 equiv), KO*t*Bu (0.05 mmol, 6 mg, 0.1 equiv), THF (1.5 mL), MeOH (0.1 mL), and [Cu–NHC] catalyst (0.5 mol %) and stirred at room temperature for 15 h. After the indicated time, the solvent was removed under reduced pressure. The sample was analyzed by ^1^H NMR (CDCl_3_, 500 MHz) and GC–MS to obtain conversion, selectivity, and yield using an internal standard and comparison with authentic samples. An analytical sample was purified by chromatography on silica gel (EtOAc/hexanes) for characterization purposes. ^1^H NMR (500 MHz, CDCl_3_) δ: 7.26 (s, 1H), 7.16 (dd, *J* = 8.1, 6.7 Hz, 2H), 7.12–7.04 (m, 3H), 7.00 (dd, *J* = 5.1, 1.9 Hz, 3H), 6.94 (dd, *J* = 6.7, 3.0 Hz, 2H), 1.20 (s, 12H). ^13^C NMR (126 MHz, CDCl_3_) δ: 143.29, 140.59, 137.16, 130.09, 129.00, 128.36, 127.98, 127.70, 126.38, 83.91, 24.94.

#### 4.2.6 General Procedure for [Ag–NHC]-Catalyzed Hydroboration of Alkynes

An oven-dried vial equipped with a stir bar was charged with diphenylacetylene (0.5 mmol, 89 mg, 1.0 equiv), bis(pinacolato)diboron (0.6 mmol, 152 mg, 1.2 equiv), KO*t*Bu (0.05 mmol, 6 mg, 0.1 equiv), MeOH (1.5 mL), and [Ag–NHC] catalyst (2.0 mol %) and stirred at 50 °C for 15 h. After the indicate time, the solvent was removed under reduced pressure. The sample was analyzed by ^1^H NMR (CDCl_3_, 500 MHz) and GC–MS to obtain conversion, selectivity, and yield using an internal standard and comparison with authentic samples.

## Supporting Information Available

Experimental details including spectra, crystallographic data, computational methods, full reference for Gaussian 09, topographical steric maps, graphical representation of frontier molecular orbitals, bond orders of [Cu(NHC)Cl] complexes, and ^1^H and ^13^C NMR spectra (PDF)
Atomic coordinates for complexes **1**, **2**, **5**, and **9** (XYZ)

## Supplementary Material

om4c00333_si_001.pdf

om4c00333_si_002.xyz
