# Supplementary material for: IPr# Complexes—Highly-Hindered, Sterically-Bulky Cu(I) and Ag(I) N-Heterocyclic Carbenes: Synthesis, Characterization, and Reactivity
Source: Organometallics. 2024 Sep 21;43(19):2305–13. doi: 10.1021/acs.organomet.4c00333 (PMC11481170; doi:10.1021/acs.organomet.4c00333)
Supplement: Supplementary file 1 — om4c00333_si_001.pdf [file om4c00333_si_001.pdf]

Supporting Information

IPr# Complexes – Highly-Hindered, Sterically-Bulky Cu(I) and Ag(I) N-Heterocyclic Carbenes: Synthesis, Characterization and Reactivity

Greta Utecht-Jarzyńska,*[,†,§] Szymon Jarzyński,[§] Md. Mahbubur Rahman,[†] Guangrong Meng,[†] Roger Lalancette,[†] Roman Szostak,[‡] and Michal Szostak*[,†]

[†]*Department of Chemistry, Rutgers University, 73 Warren Street, Newark, NJ 07102, USA*
[§] *University of Lodz, Faculty of Chemistry, Tamka 12, 91-403 Łódź, Poland*
[‡]*Department of Chemistry, Wroclaw University, F. Joliot-Curie 14, Wroclaw 50-383, Poland*

greta.utecht@chemia.uni.lodz.pl; michal.szostak@rutgers.edu

Table of Contents S1

Crystallographic Studies S2
Computational Methods S12
Bond Orders of [Cu(NHC)Cl] Complexes S16
^{1}H and ^{13}C NMR Spectra S17

Corresponding Author:
Prof. Dr. M. Szostak
Department of Chemistry, Rutgers University
73 Warren Street, Newark, NJ 07102, United States
E-mail: michal.szostak@rutgers.edu

Details of Crystal Structure Analysis

Crystallographic information for all the compounds are given in Tables S1-S4 in the Supporting Information. All compounds were colorless single crystals. Full datasets were collected using graphite-monochromated CuKα radiation (λ = 1.54178 Å) on a Bruker SMART APEX2 single crystal diffractometer. X-rays were provided by a fine-focus sealed X-ray tube operated at 48kV and 30mA. Lattice constants were all determined using the Bruker SAINT software package using all available reflections (after data collection, ORTEP files, see Figures S1-S4).

All data were corrected for absorption by measuring the faces of each crystal and doing a numerical absorption correction. The Bruker software package SHELXTL-2014 was used to solve all the structures using the direct methods technique and difference electron density maps. All stages of weighted full-matrix least-squares refinement were conducted using F_o^2 data with the same software package. The final structural model for each compound was refined using anisotropic thermal parameters for all non-hydrogen atoms; all the H atoms were located in difference maps but were placed in geometrically idealized positions and allowed to “ride” on their parent C, O or N atoms, with bond lengths of 0.95, 1.00, 0.99, 0.98, and 0.84 Å for aromatic, methine, methylene, methyl, and hydroxyl, respectively. The isotropic thermal parameters for these H atoms were fixed to be 1.2 times the U_{iso} for C or N and 1.5 times the U_{iso} for O.

Figure S1. ORTEP Structure of [Cu(IPr$^{\#}$)Cl] (**1**) (50% ellipsoids). (Crystallographic data has been deposited with the Cambridge Crystallographic Data Center as supplementary publication no. CCDC 2373450).

Front View:

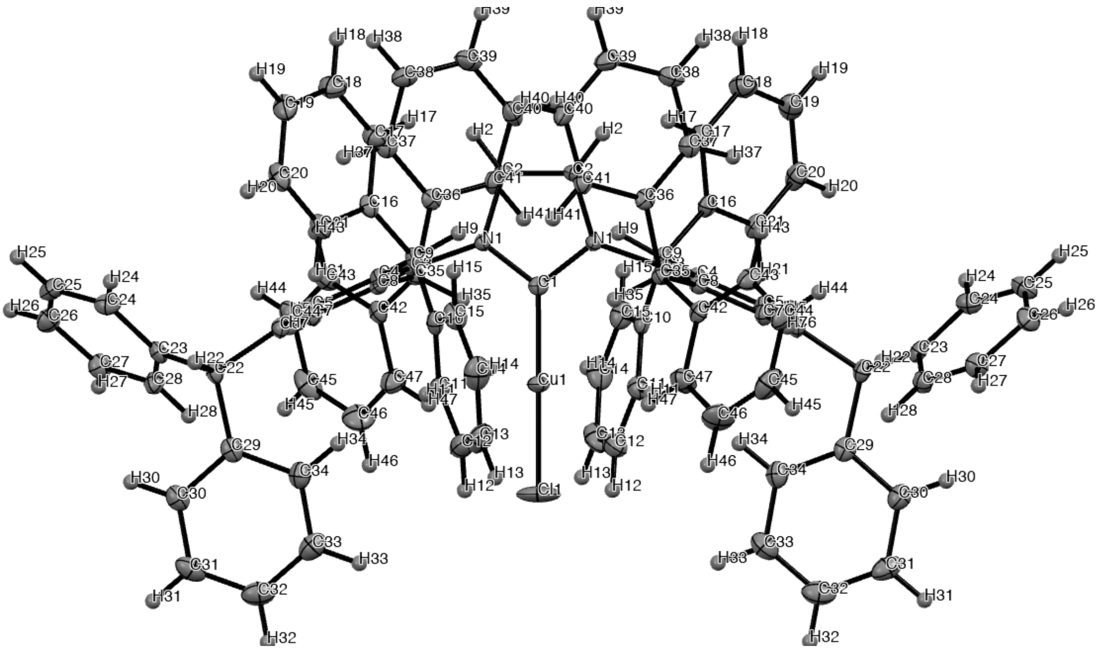

Side View:

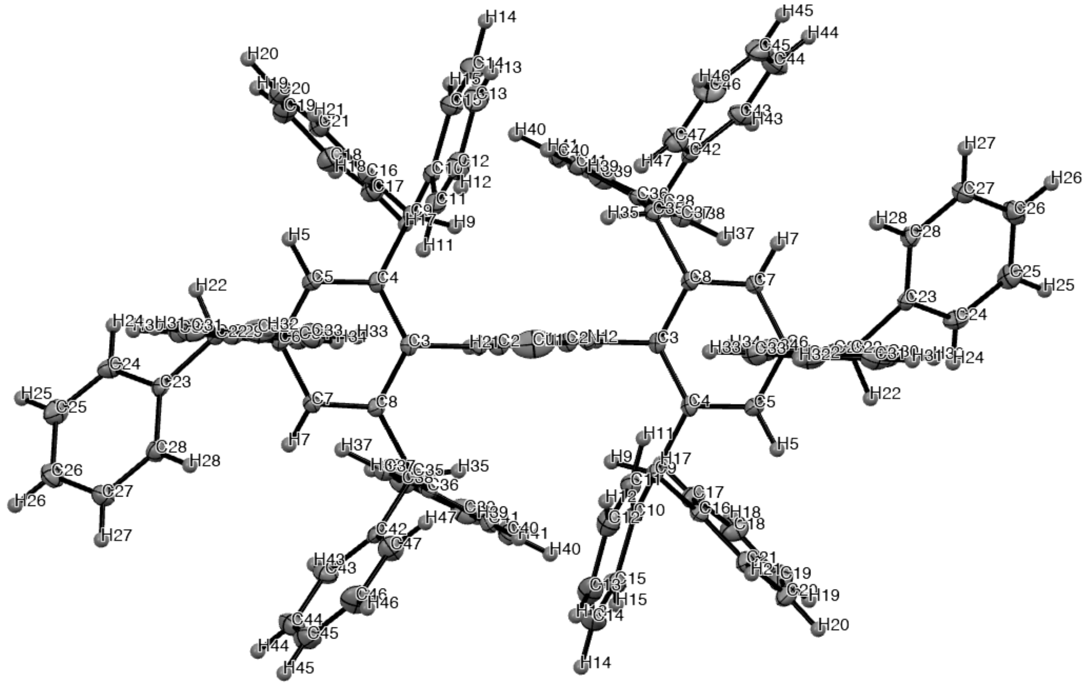

Selected bond lengths [Å], bond angle [°] and dihedral angles [°] (**1**): Cu1–Cl1, 2.109(9); Cu1–C1, 1.885(3); C1–N1, 1.355(2); N1–C3, 1.443(2); Cl1–Cu1–C1, 180.00; Cu1–C1–N1, 127.91(11); C1–N1–C3, 122.48(16); Cu1–C1–N1–C3, 4.40; C3–N1–N1–C3, 21.98.

Table S1. Crystal Data and Structure Refinement Summaries for [Cu(IPr$^{\#}$)Cl] (**1**).

Compound	**1**
Chemical formula	$C_{93}H_{72}ClCuN_2$
M_r	1316.51
Crystal system, space group	Monoclinic, *C*2/*c*
Temperature (K)	100 (2)
a, *b*, *c* (Å)	29.241 (4), 9.6772 (13), 26.262 (4)
α, β, γ (°)	90, 113.952 (7), 90
V (Å^3)	6791.3 (16)
Z	4
Radiation type	Cu *K*α
μ (mm^{-1})	1.21
Crystal size (mm)	0.46 × 0.21 × 0.16
Diffractometer	Bruker *SMART* CCD Apex-II area-detector
Absorption correction	Numerical<br>*SADABS* (Sheldrick, 2008a)
T_{min}, T_{max}	0.557, 0.861
No. of measured, independent and observed [$I > 2\sigma(I)$] reflections	24383, 5724, 5167
R_{int}	0.064
$(\sin \theta/\lambda)_{max}$ (Å^{-1})	0.606
$R[F^2 > 2\sigma(F^2)]$, $wR(F^2)$, S	0.051, 0.142, 1.09
No. of reflections	5724
No. of parameters	439
No. of restraints	0
H-atom treatment	H-atom parameters constrained
$\Delta\rho_{max}$, $\Delta\rho_{min}$ (e Å^{-3})	1.32, −0.87

Computer programs: *APEX* 2 (Bruker, 2006), *APEX* 2, *SAINT* (Bruker, 2005), *SHELXL2014*/7 (Sheldrick, 2014), *SHELXTL*.

Figure S2. ORTEP Structure of [Cu(BIAN-IPr$^{\#}$)Cl] (**2**) (50% ellipsoids). (Crystallographic data has been deposited with the Cambridge Crystallographic Data Center as supplementary publication no. CCDC 2373451).

Front View:

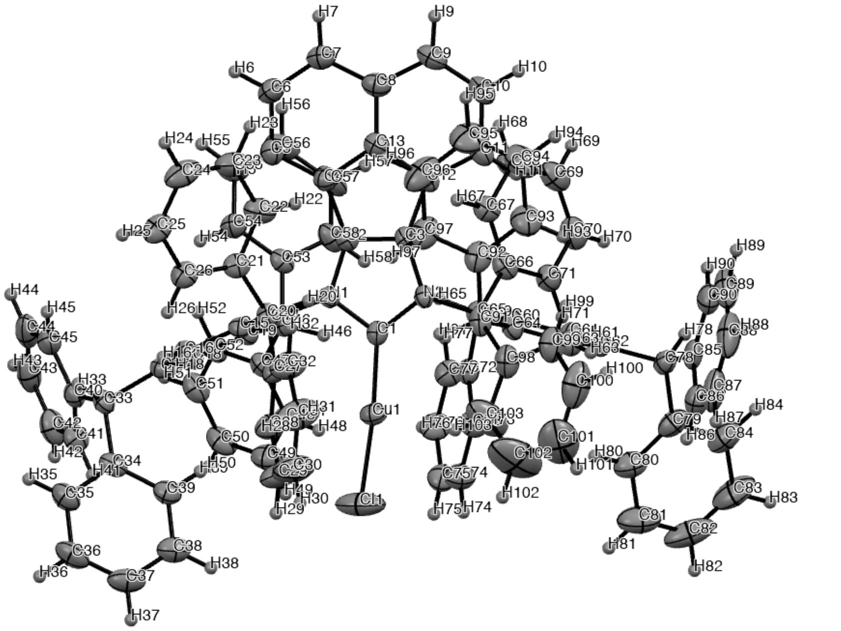

Side View:

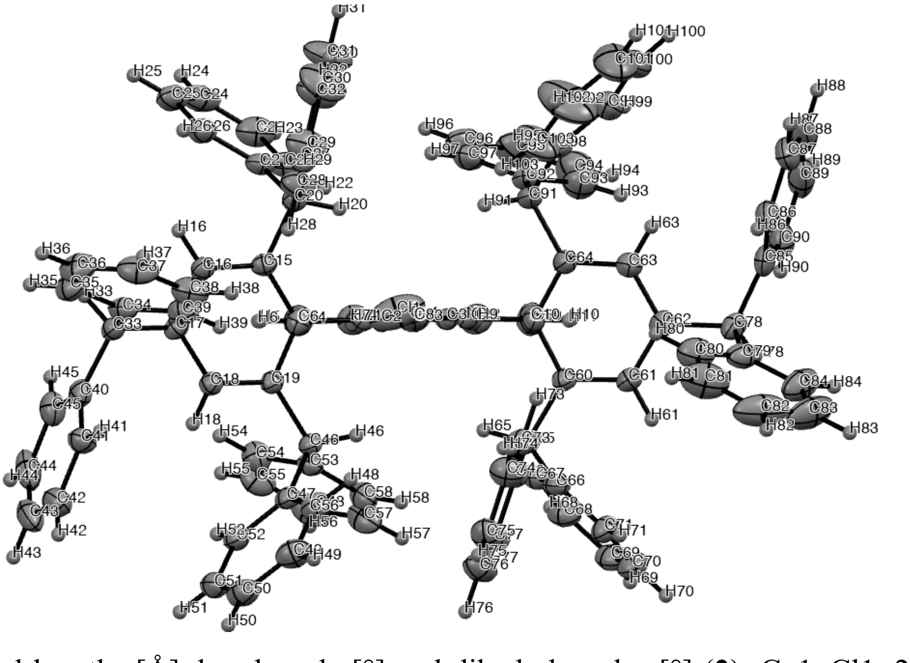

Selected bond lengths [Å], bond angle [°] and dihedral angles [°] (**2**): Cu1–Cl1, 2.0898(13); Cu1–C1, 1.869(3); C1–N1, 1.375(4); C1–N2, 1.368(4); N1–C14, 1.434(4), N2–C59, 1.448(4); Cl1–Cu1–C1, 174.57(11); Cu1–C1–N1, 124.2(2); Cu1–C1–N2, 130.7(2); C1–N1–C14, 122.4(3); C1–N2–C59, 124.4(3)0; Cu1–C1–N1–C14, 2.44; Cu1–C1–N2–C59, 4.21; C14–N1–N2–C59, 4.79.

Table S2. Crystal Data and Structure Refinement Summaries for [Cu(BIAN-IPr$^{\#}$)Cl] (**2**).

Compound	**2**
Chemical formula	$C_{103}H_{76}ClCuN_2$, 3(CH_2Cl_2)
M_r	1695.42
Crystal system, space group	Triclinic, *P*-1
Temperature (K)	100 (2)
a, *b*, *c* (Å)	14.8225 (2), 16.8908 (2), 17.9791 (2)
α, β, γ (°)	95.562 (1), 95.106 (1), 105.477 (1)
V (Å^3)	4286.59 (9)
Z	2
Radiation type	Cu *K*α
μ (mm^{-1})	2.76
Crystal size (mm)	0.22 × 0.13 × 0.11
Diffractometer	XtaLAB Synergy, Dualflex, HyPix
Absorption correction	CrysAlisPro 1.171.42.96a (Rigaku Oxford Diffraction, 2023)
No. of measured, independent and observed [$I > 2\sigma(I)$] reflections	93903, 16306, 13840
R_{int}	0.037
$(\sin \theta/\lambda)_{max}$ (Å^{-1})	0.614
$R[F^2 > 2\sigma(F^2)]$, $wR(F^2)$, S	0.107, 0.366, 1.70
No. of reflections	16306
No. of parameters	1045
No. of restraints	18
H-atom treatment	H-atom parameters constrained
$\Delta\rho_{max}$, $\Delta\rho_{min}$ (e Å^{-3})	2.07, −2.06

Computer programs: *SHELXL2018*/3 (Sheldrick, 2018).

Figure S3. ORTEP Structure of [Ag(IPr$^{\#}$)Cl] (**3**) (50% ellipsoids). (Crystallographic data has been deposited with the Cambridge Crystallographic Data Center as supplementary publication no. CCDC 2373455).

Front View:

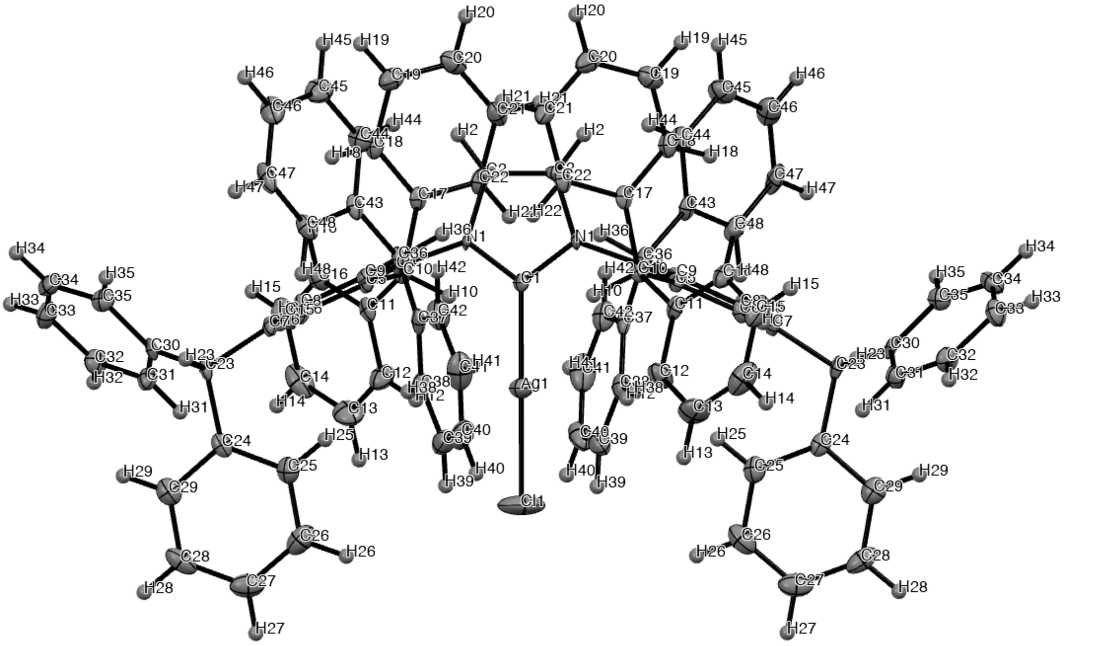

Front View:

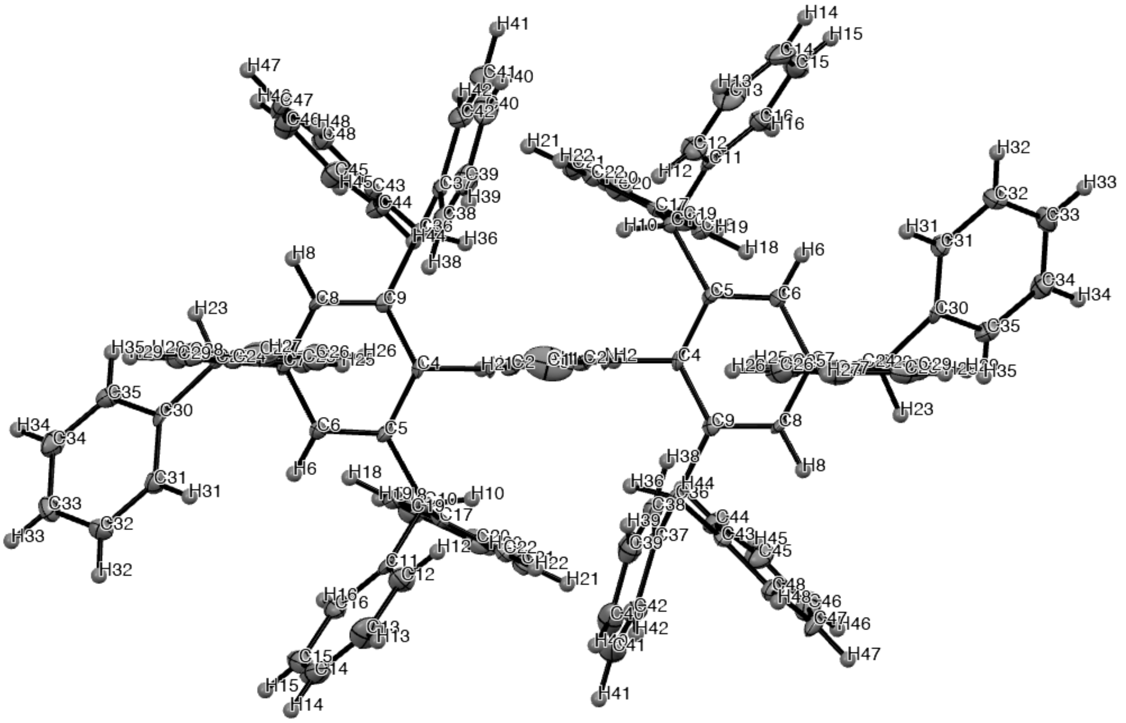

Selected bond lengths [Å], bond angle [°] and dihedral angles [°] (**3**): Ag1–Cl1, 2.2983(17); Ag1–C1, 2.071(5); C1–N1, 1.348(4); N1–C4, 1.448(4); Cl1–Ag1–C1, 180.0; Ag1–C1–N1, 127.3(2); C1–N1–C4, 122.9(3); Ag1–C1–N1–C4, 4.82,; C4–N1–N1–C4, 23.64.

Table S3. Crystal Data and Structure Refinement Summaries for [Ag(IPr#)Cl] (**3**).

Compound	**3**
Chemical formula	$C_{93}H_{72}AgClN_2$
M_r	1360.84
Crystal system, space group	Monoclinic, *C*2/*c*
Temperature (K)	100 (2)
a, *b*, *c* (Å)	29.3136 (4), 9.8089 (1), 26.1454 (4)
α, β, γ (°)	90, 114.091 (1), 90
V (Å^3)	6862.89 (16)
Z	4
Radiation type	Cu *K*α
μ (mm^{-1})	3.10
Crystal size (mm)	0.26 × 0.08 × 0.07
Diffractometer	Bruker *SMART* CCD Apex-II area-detector
Absorption correction	Numerical<br>*SADABS* 2016/2: Krause, L., Herbst-Irmer, R., Sheldrick G.M. & Stalke D., J. Appl. Cryst. 48 (2015) 3-10
T_{min}, T_{max}	0.659, 0.853
No. of measured, independent and observed [$I > 2\sigma(I)$] reflections	31281, 5995, 4781
R_{int}	0.065
$(\sin \theta/\lambda)_{max}$ (Å^{-1})	0.606
$R[F^2 > 2\sigma(F^2)]$, $wR(F^2)$, S	0.051, 0.138, 1.04
No. of reflections	5995
No. of parameters	439
No. of restraints	0
H-atom treatment	H-atom parameters constrained
$\Delta\rho_{max}$, $\Delta\rho_{min}$ (e Å^{-3})	0.70, −1.07

Computer programs: *APEX* 2 (Bruker, 2006), *APEX* 2, *SAINT* (Bruker, 2005), *SHELXL2014*/7 (Sheldrick, 2014), *SHELXTL*.

Figure S4. ORTEP Structure of [Ag(BIAN-IPr$^{\#}$)Cl] (**4**) (50% ellipsoids). (Crystallographic data has been deposited with the Cambridge Crystallographic Data Center as supplementary publication no. CCDC 2373458).

Front View:

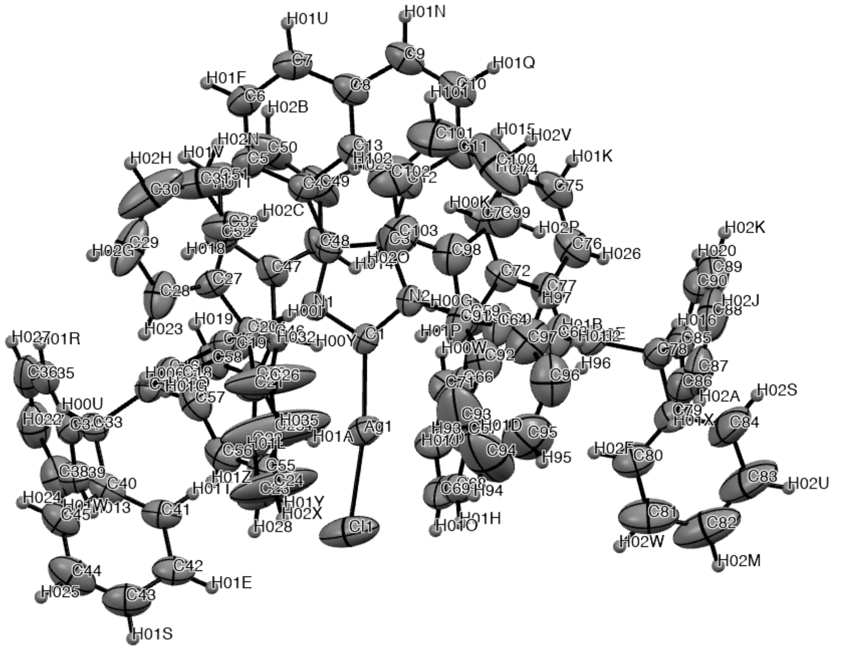

Side View:

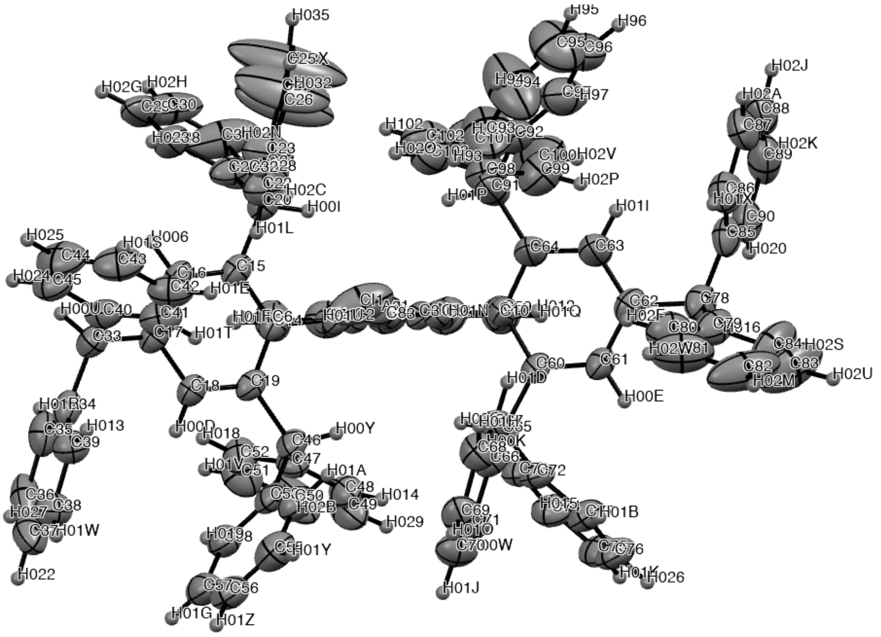

Selected bond lengths [Å], bond angle [°] and dihedral angles [°] (**4**): Ag1–Cl1, 2.308(2); Ag1–C1, 2.078(5); C1–N1, 1.377(6); C1–N2, 1.343(6); N1–C14, 1.435(6), N2–C59, 1.437(6); Cl1–Ag1–C1, 172.31(14); Ag1–C1–N1, 122.8(3); Ag1–C1–N2, 130.9(4); C1–N1–C14, 122.2(4); C1–N2–C59, 124.6(4); Ag1–C1–N1–C14, 4.95; Ag1–C1–N2–C59, 2.80; C14–N1–N2–C59, 5.09.

Table S4. Crystal Data and Structure Refinement Summaries for [Ag(BIAN-IPr#)Cl] (**4**).

Compound	**4**
Chemical formula	$C_{103}H_{76}AgClN_2$
M_r	1484.97
Crystal system, space group	Triclinic, *P*-1
Temperature (K)	100(2)
a, *b*, *c* (Å)	14.9146 (2), 16.6595 (2), 18.0469 (2)
α, β, γ (°)	94.768 (1), 94.443 (1), 104.654 (1)
V (Å^3)	4300.61 (9)
Z	2
Radiation type	Cu *K*α
μ (mm^{-1})	2.52
Crystal size (mm)	0.21 × 0.14 × 0.12
Diffractometer	XtaLAB Synergy, Dualflex, HyPix
Absorption correction	CrysAlisPro 1.171.42.96a (Rigaku Oxford Diffraction, 2023)
No. of measured, independent and observed [$I > 2\sigma(I)$] reflections	78329, 16308, 13656
R_{int}	0.037
$(\sin \theta/\lambda)_{max}$ (Å^{-1})	0.614
$R[F^2 > 2\sigma(F^2)]$, $wR(F^2)$, S	0.107, 0.354, 1.61
No. of reflections	16308
No. of parameters	958
No. of restraints	108
H-atom treatment	H-atom parameters constrained
$\Delta\rho_{max}$, $\Delta\rho_{min}$ (e Å^{-3})	4.34, −1.03

Computer programs: *SHELXL2018*/3 (Sheldrick, 2018).

Figure S5. (A-D) Topographical Steric Maps of [Cu(NHC)Cl] and [Ag(NHC)Cl] Complexes Calculated from X-ray Single Crystal Structures, Showing %V_{bur} per Quadrant.

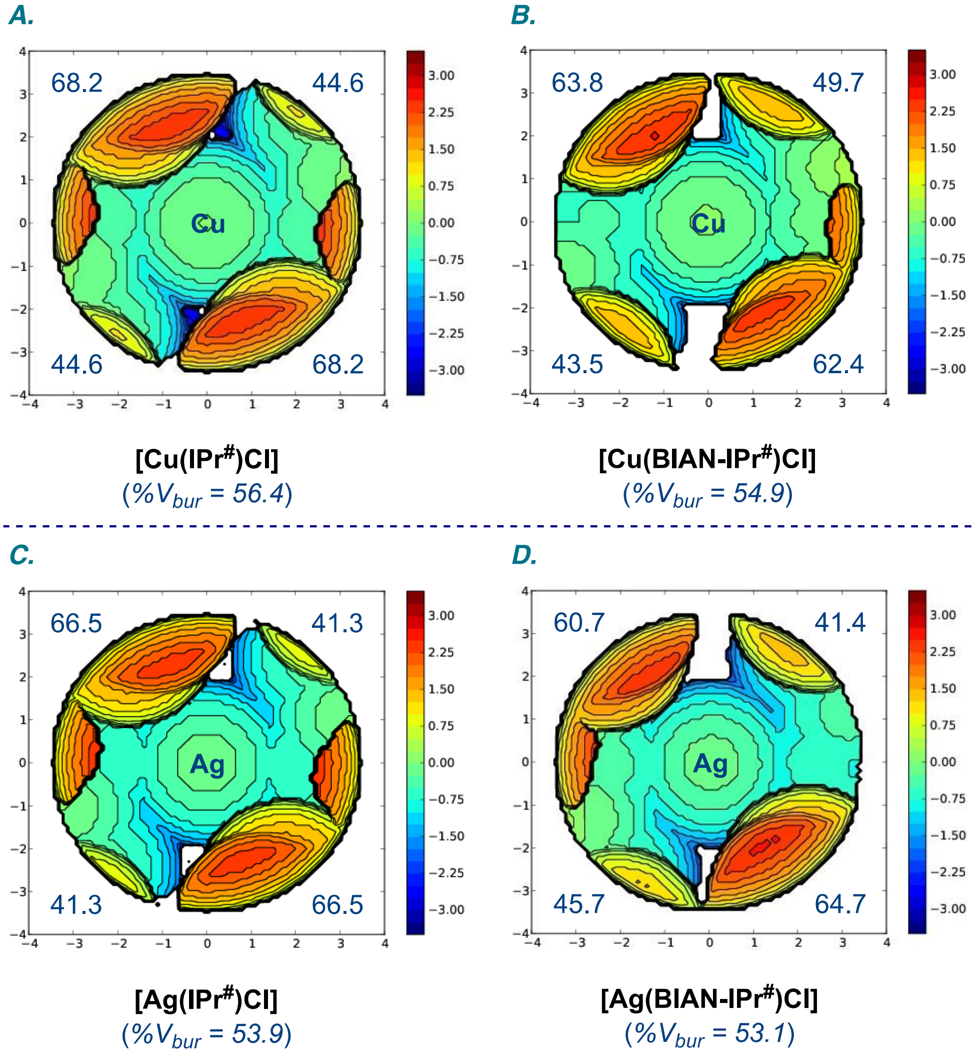

Computational Methods

Computational Methods. All the calculations were performed using Gaussian 09 suite of programs. All of the geometry optimizations were performed at the B3LYP level of theory with the 6-311++G(d,p) basis set. For geometry optimizations, we employed the X-ray structures of [Cu(IPr$^{\#}$)Cl], [Cu(BIAN-IPr$^{\#}$)Cl], [Ag(IPr$^{\#}$)Cl] and [Ag(BIAN-IPr$^{\#}$)Cl] complexes as the starting geometry and performed full optimization. The absence of imaginary frequencies was used to characterize the structures as minima on the potential energy surface. All of the optimized geometries were verified as minima (no imaginary frequencies). NBO calculations were performed at the DFT/B3LYP level using NBO program implemented in Gaussian software package. Wiberg bond indices were calculated by the NBO method (*J. Mol. Struc. Theochem* **2008**, *870*, 1). Energetic parameters were calculated under standard conditions (298.15 K and 1 atm). Structural representations were generated using CYLview software (Legault, C. Y. CYL view version 1.0 BETA, University of Sherbrooke). All other representations were generated using Gauss View (GaussView, version 5, Dennington, R.; Keith, T.; Millam, J. Semichem Inc., Shawnee Mission, KS, 2009) or ChemCraft software (Andrienko, G. L. ChemCraft version b562a, https://www.chemcraftprog.com/).

Full Reference for Gaussian 09

Gaussian 09, Revision D.01, Frisch, M. J.; Trucks, G. W.; Schlegel, H. B.; Scuseria, G. E.; Robb, M. A.; Cheeseman, J. R.; Scalmani, G.; Barone, V.; Mennucci, B.; Petersson, G. A.; Nakatsuji, H.; Caricato, M.; Li, X.; Hratchian, H. P.; Izmaylov, A. F.; Bloino, J.; Zheng, G.; Sonnenberg, J. L.; Hada, M.; Ehara, M.; Toyota, K.; Fukuda, R.; Hasegawa, J.; Ishida, M.; Nakajima, T.; Honda, Y.; Kitao, O.; Nakai, H.; Vreven, T.; Montgomery, J. A., Jr.; Peralta, J. E.; Ogliaro, F.; Bearpark, M.; Heyd, J. J.; Brothers, E.; Kudin, K. N.; Staroverov, V. N.; Kobayashi, R.; Normand, J.; Raghavachari, K.; Rendell, A.; Burant, J. C.; Iyengar, S. S.; Tomasi, J.; Cossi, M.; Rega, N.; Millam, M. J.; Klene, M.; Knox, J. E.; Cross, J. B.; Bakken, V.; Adamo, C.; Jaramillo, J.; Gomperts, R.; Stratmann, R. E.; Yazyev, O.; Austin, A. J.; Cammi, R.; Pomelli, C.; Ochterski, J. W.; Martin, R. L.; Morokuma, K.; Zakrzewski, V. G.; Voth, G. A.; Salvador, P.; Dannenberg, J. J.; Dapprich, S.; Daniels, A. D.; Farkas, Ö.; Foresman, J. B.; Ortiz, J. V.; Cioslowski, J.; Fox, D. J. Gaussian, Inc., Wallingford CT, 2009.

Figure S6. (A-D) Topographical Steric Maps of [Cu(IPr$^{\#}$)Cl], [Cu(IPr*)Cl], [Cu(BIAN-IPr$^{\#}$)Cl] and [Cu(BIAN-IPr*)Cl] Complexes Calculated at the B3LYP 6-311++g(d,p) Level.

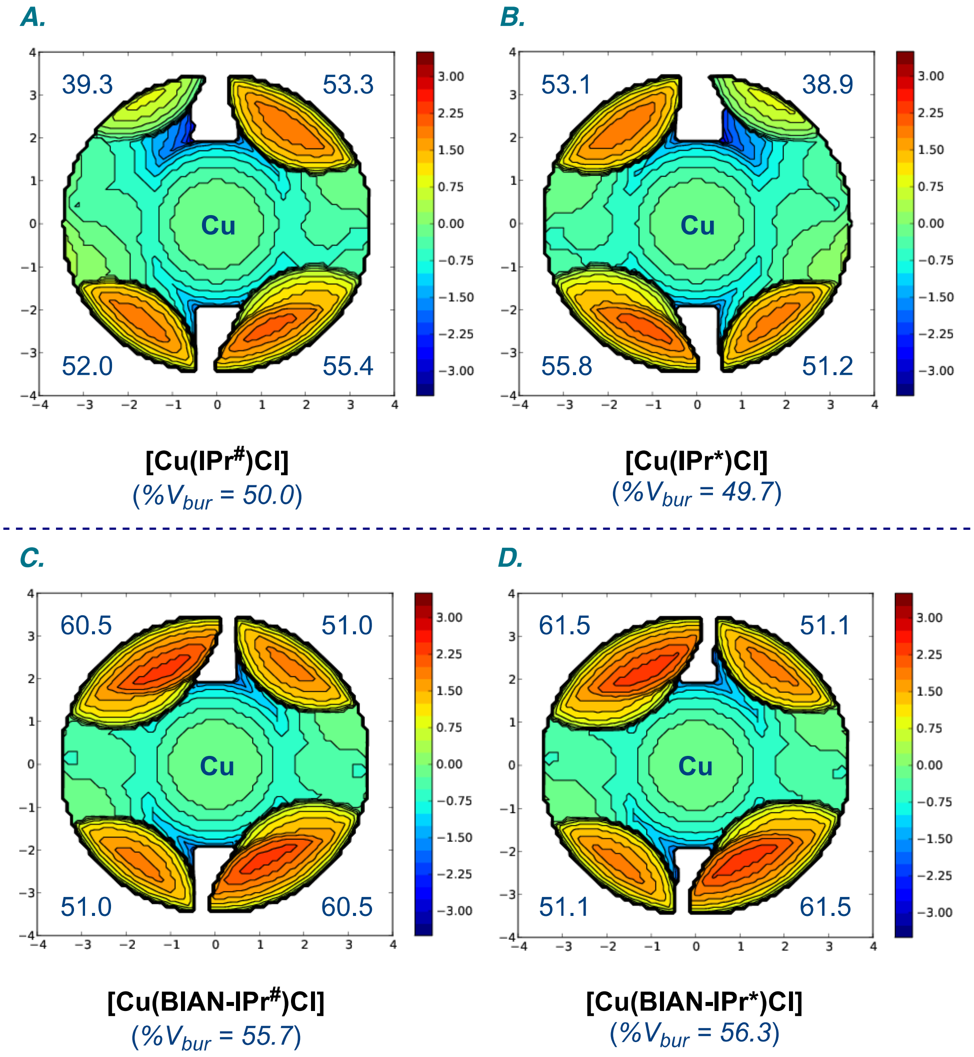

Figure S7. (A-D) Graphical Representation of Frontier Molecular Orbitals of [Cu(IPr#)Cl], [Cu(IPr*)Cl], [Cu(BIAN-IPr#)Cl] and [Cu(BIAN-IPr*)Cl] Complexes Calculated at the B3LYP 6-311++g(d,p) Level.

A. [Cu(IPr#)Cl]

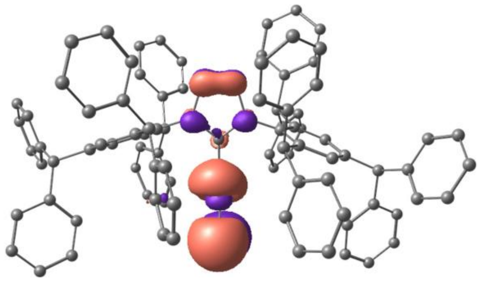

HOMO (-6.15 *eV*)

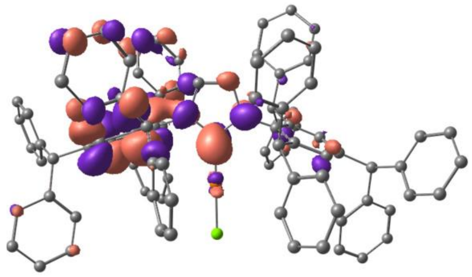

LUMO (-1.22 *eV*)

B. [Cu(BIAN-IPr#)Cl]

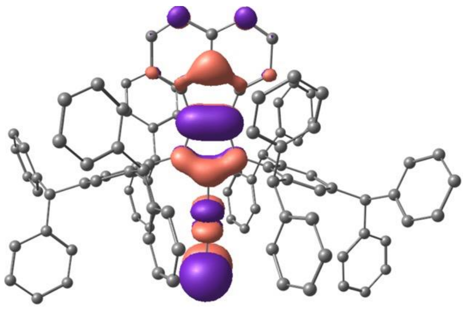

HOMO (-5.86 *eV*)

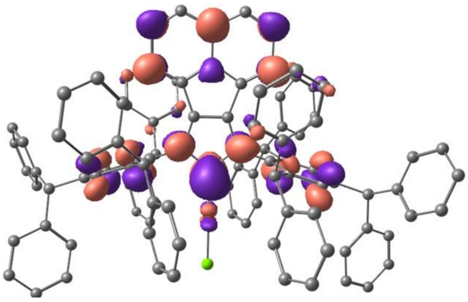

LUMO+1 (-1.24 *eV*)

C. [Cu(IPr*)Cl]

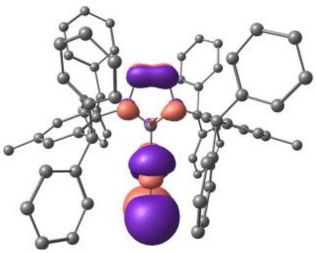

HOMO (-6.11 *eV*)

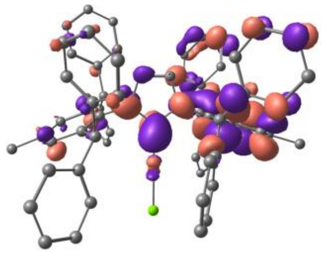

LUMO (-1.20 *eV*)

D. [Cu(BIAN-IPr*)Cl]

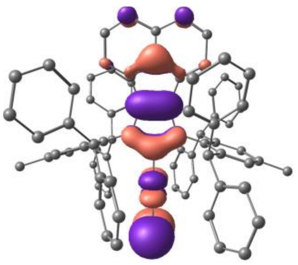

HOMO (-5.85 *eV*)

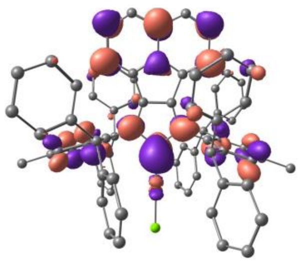

LUMO+1 (-1.23 *eV*)

Table S5. Bond Orders of [Cu(NHC)Cl] Complexes.

Entry	Structure	Cu–C$_{(carbene)}$	Cu–Cl
1	[Cu(IPr$^{\#}$)Cl]	0.4488	0.5433
2	[Cu(BIAN-IPr$^{\#}$)Cl]	0.4506	0.5334
3	[Cu(IPr*)Cl]	0.4508	0.5480
4	[Cu(BIAN-IPr*)Cl]	0.4510	0.5347

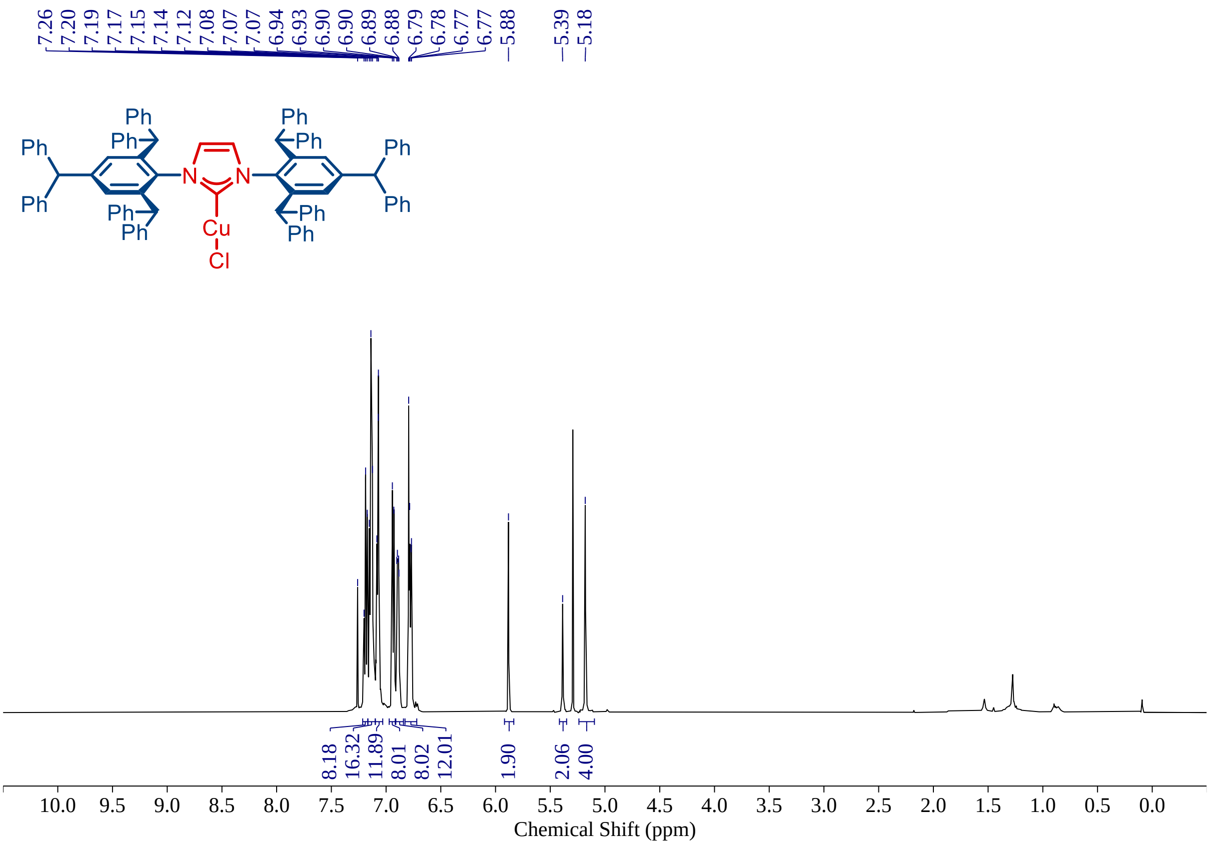

Figure S8. ^{1}H NMR (500 MHz, $CDCl_3$) Spectrum of Compound **1**.

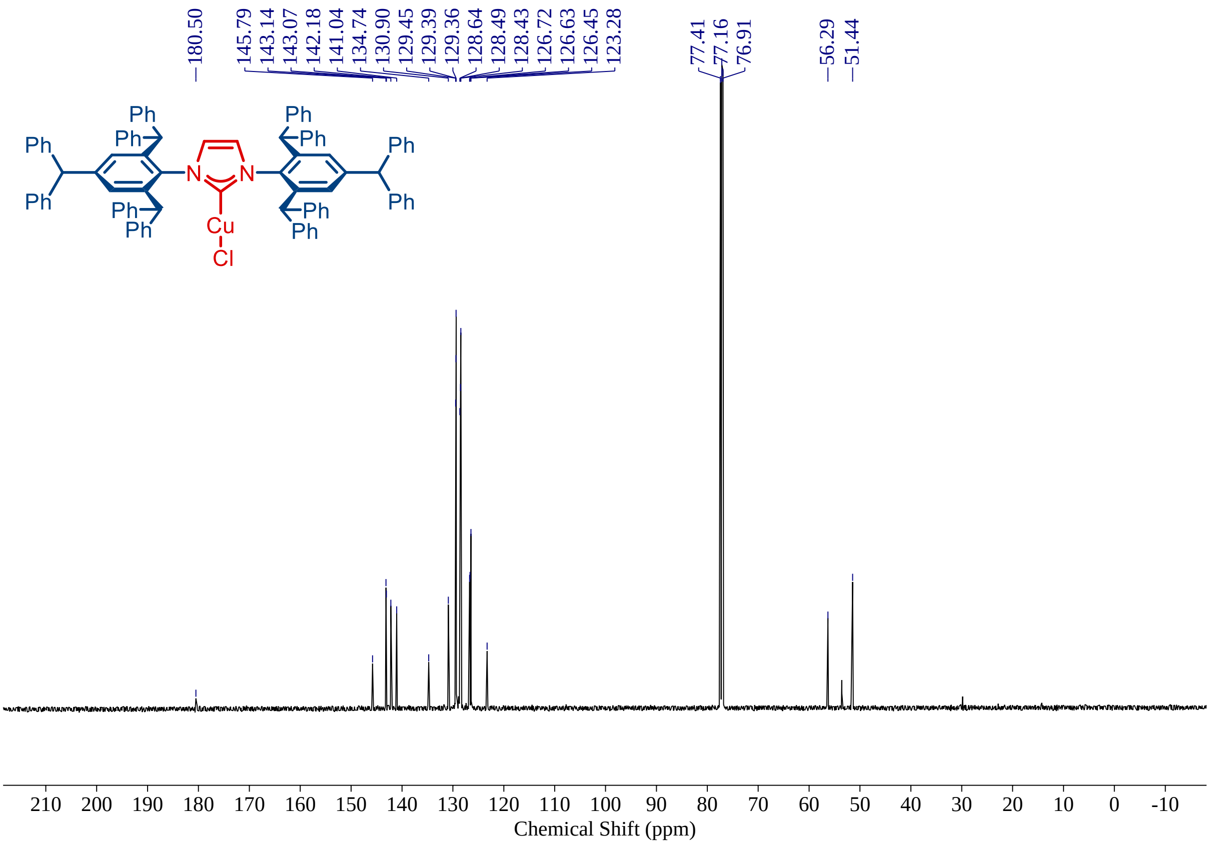

Figure S9. ^{13}C NMR (126 MHz, $CDCl_3$) Spectrum of Compound **1**.

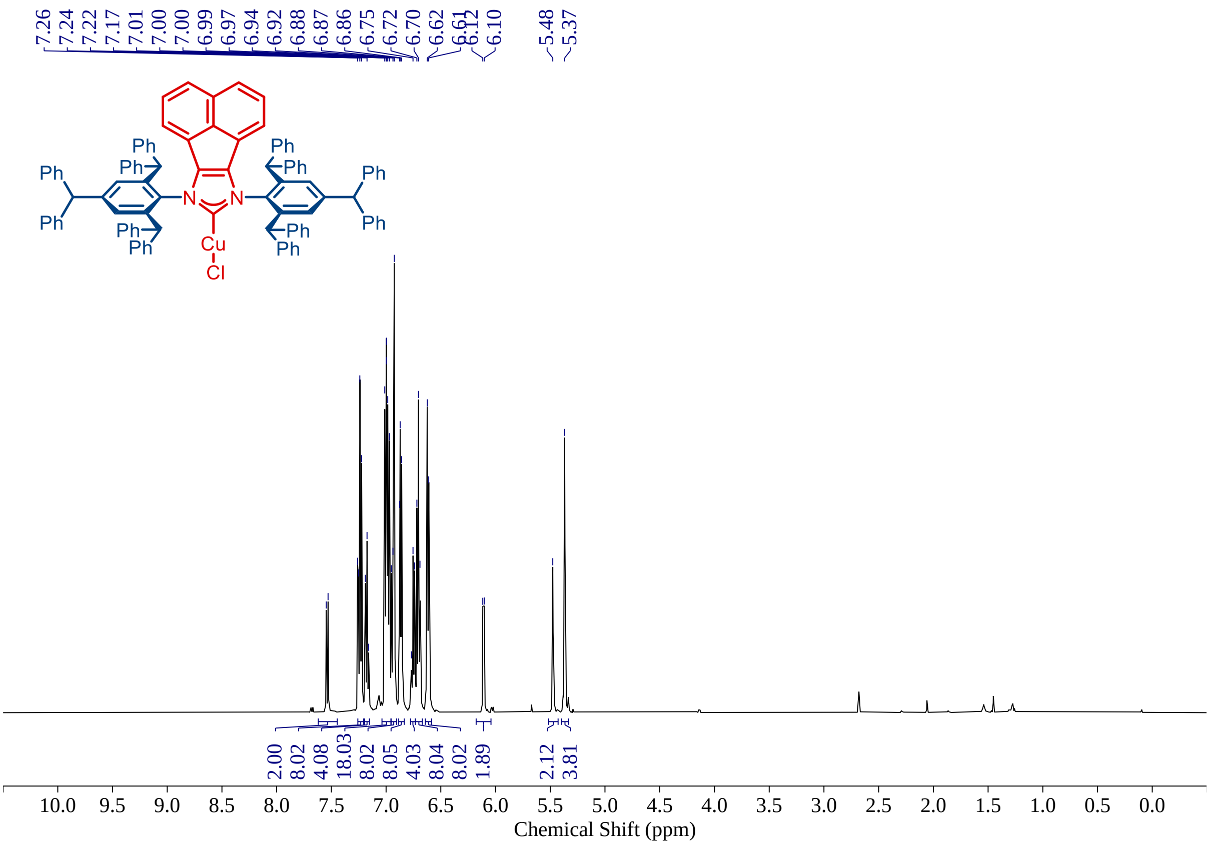

Figure S10. ^{1}H NMR (500 MHz, $CDCl_3$) Spectrum of Compound **2**.

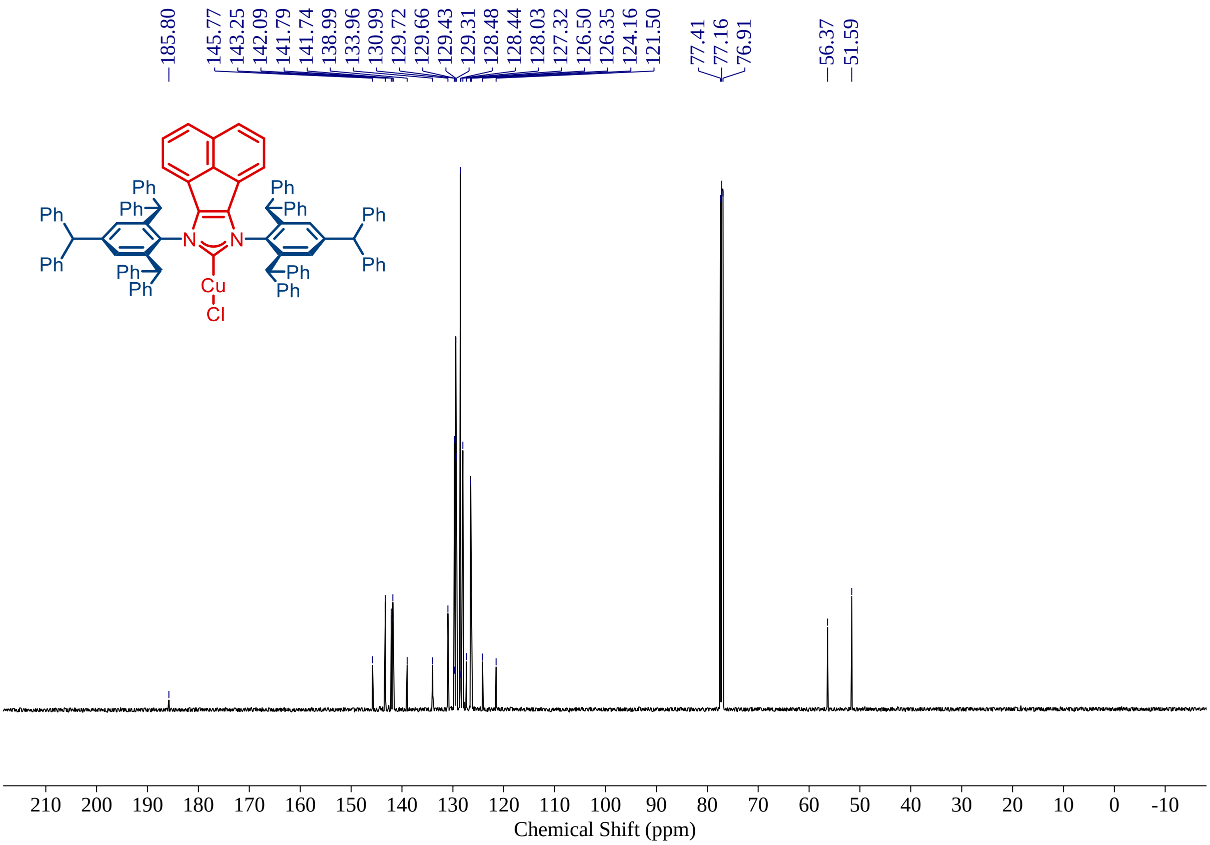

Figure S11. ^{13}C NMR (126 MHz, $CDCl_3$) Spectrum of Compound **2**.

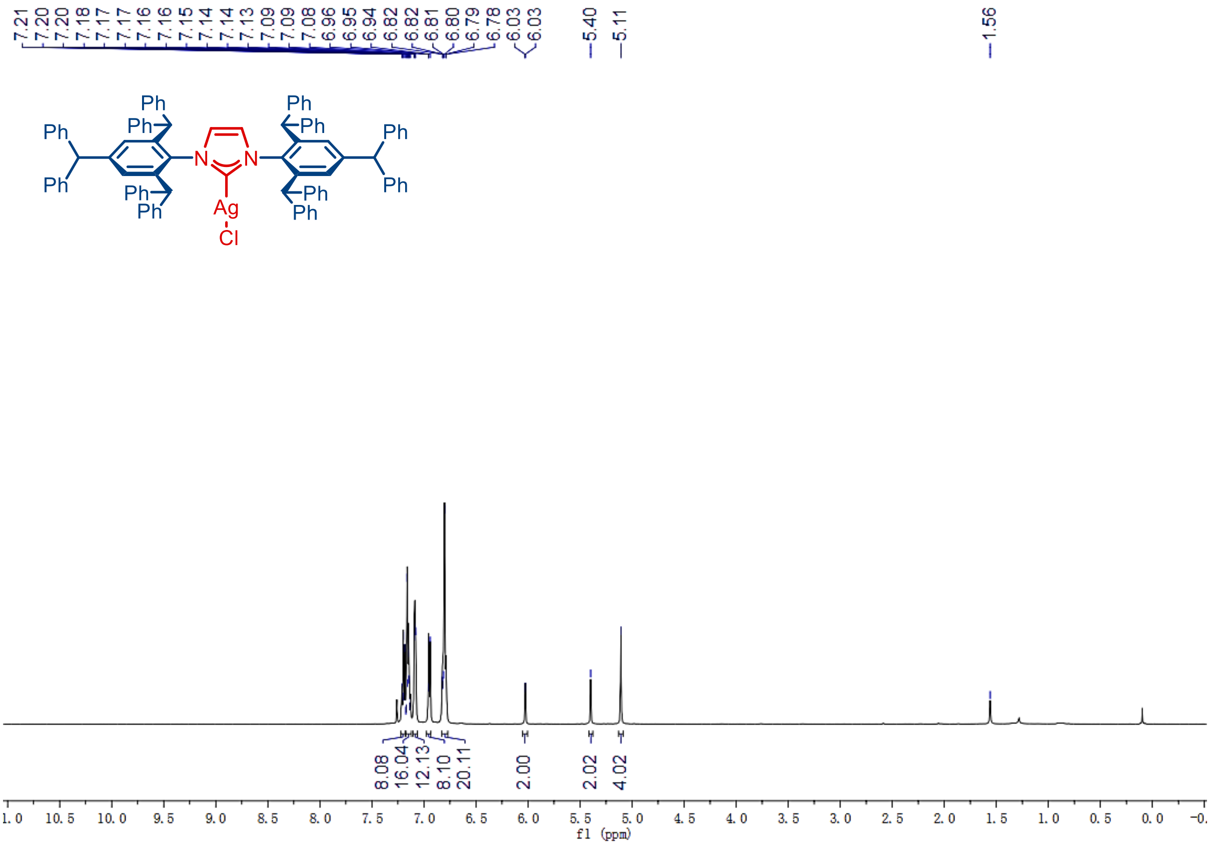

Figure S12. ^{1}H NMR (500 MHz, $CDCl_3$) Spectrum of Compound **3**.

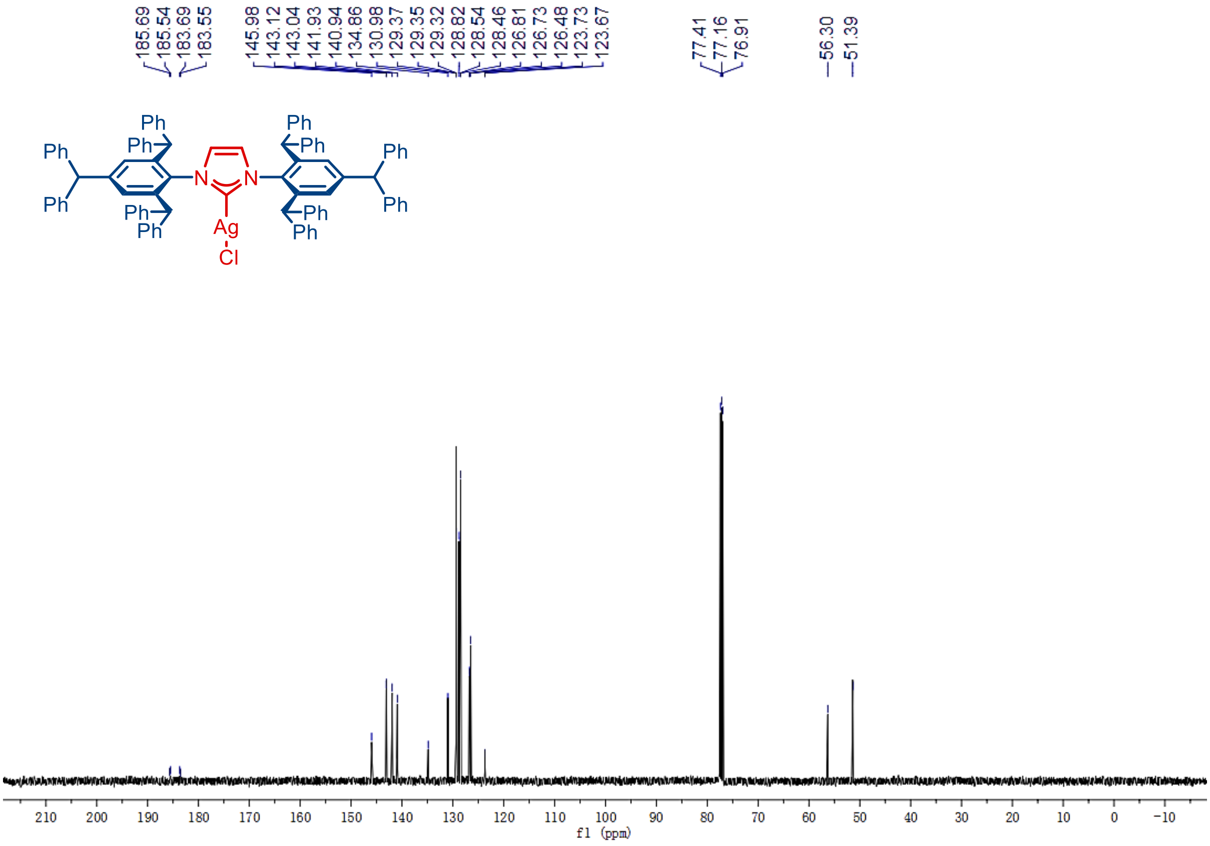

Figure S13. ^{13}C NMR (126 MHz, $CDCl_3$) Spectrum of Compound **3**.

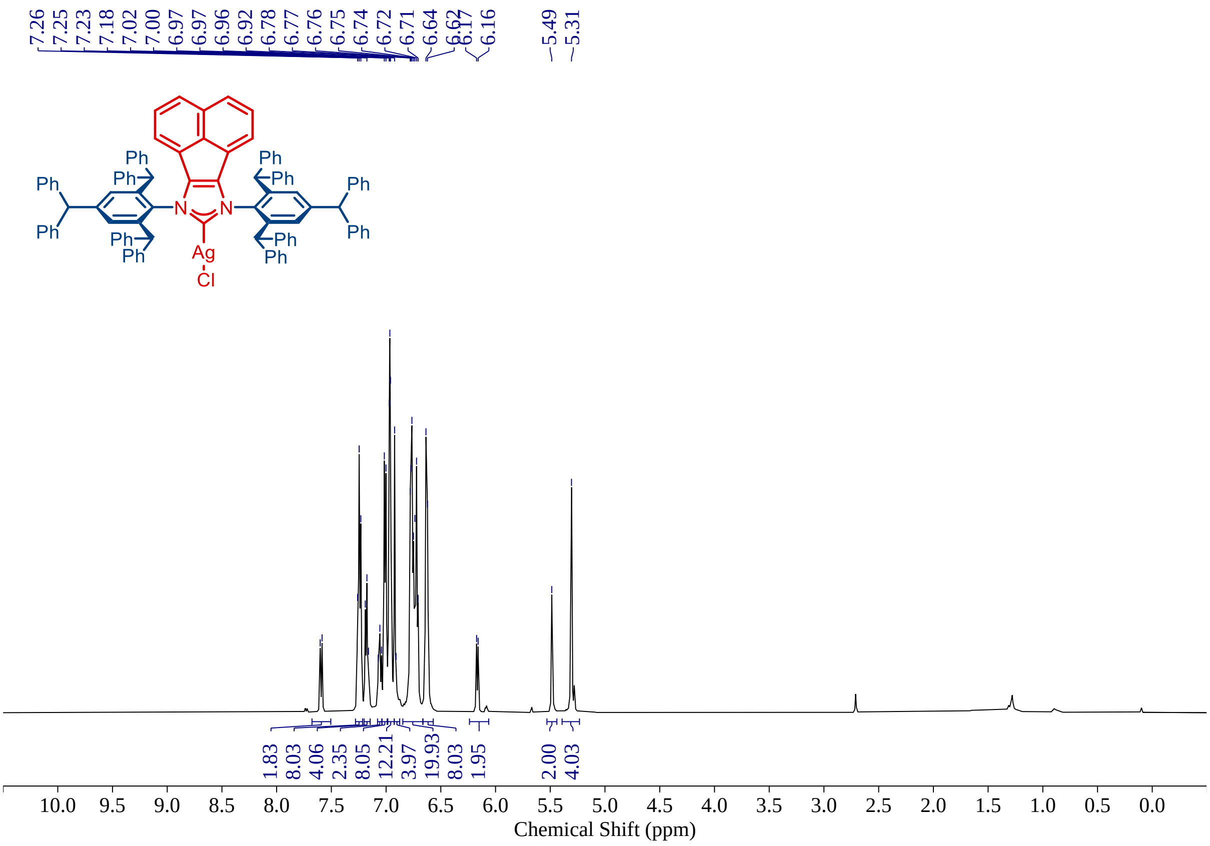

Figure S14. ^{1}H NMR (500 MHz, $CDCl_3$) Spectrum of Compound **4**.

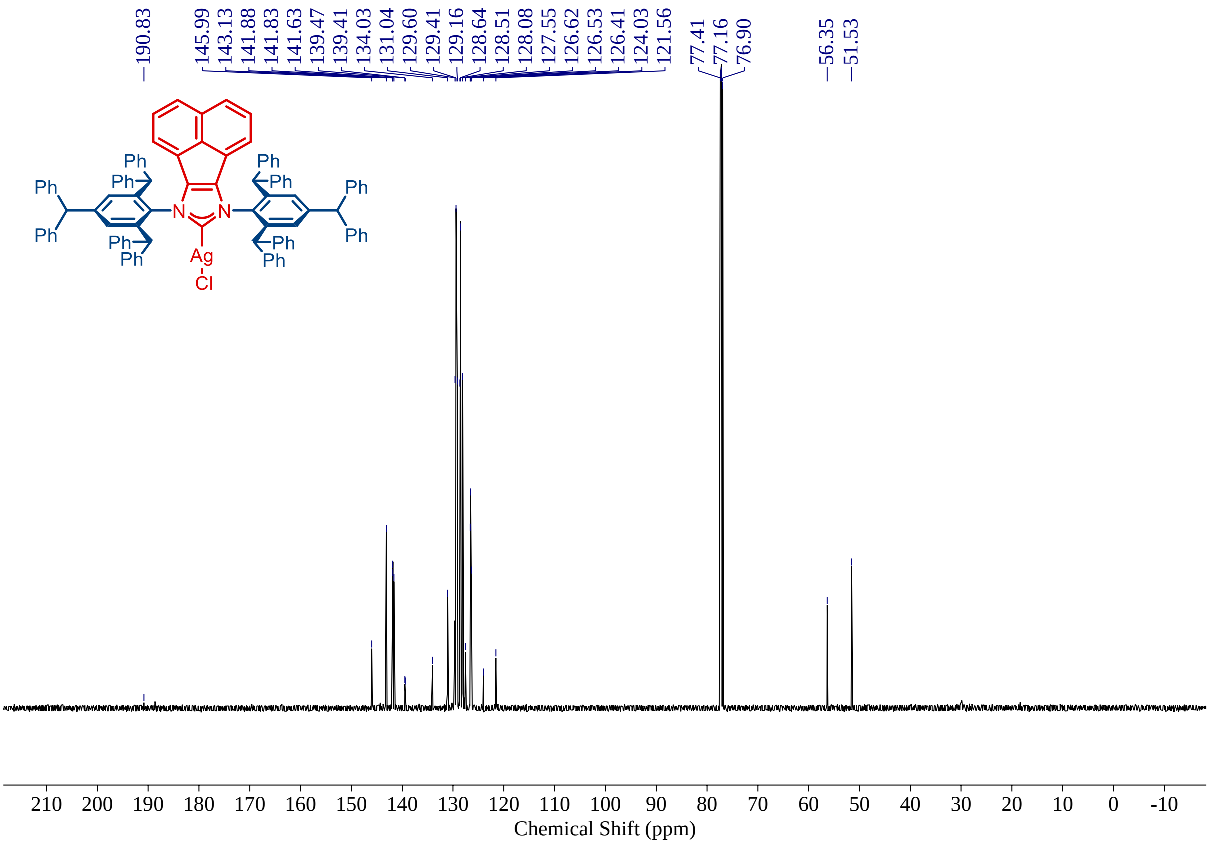

Figure S15. ^{13}C NMR (126 MHz, $CDCl_3$) Spectrum of Compound **4**.

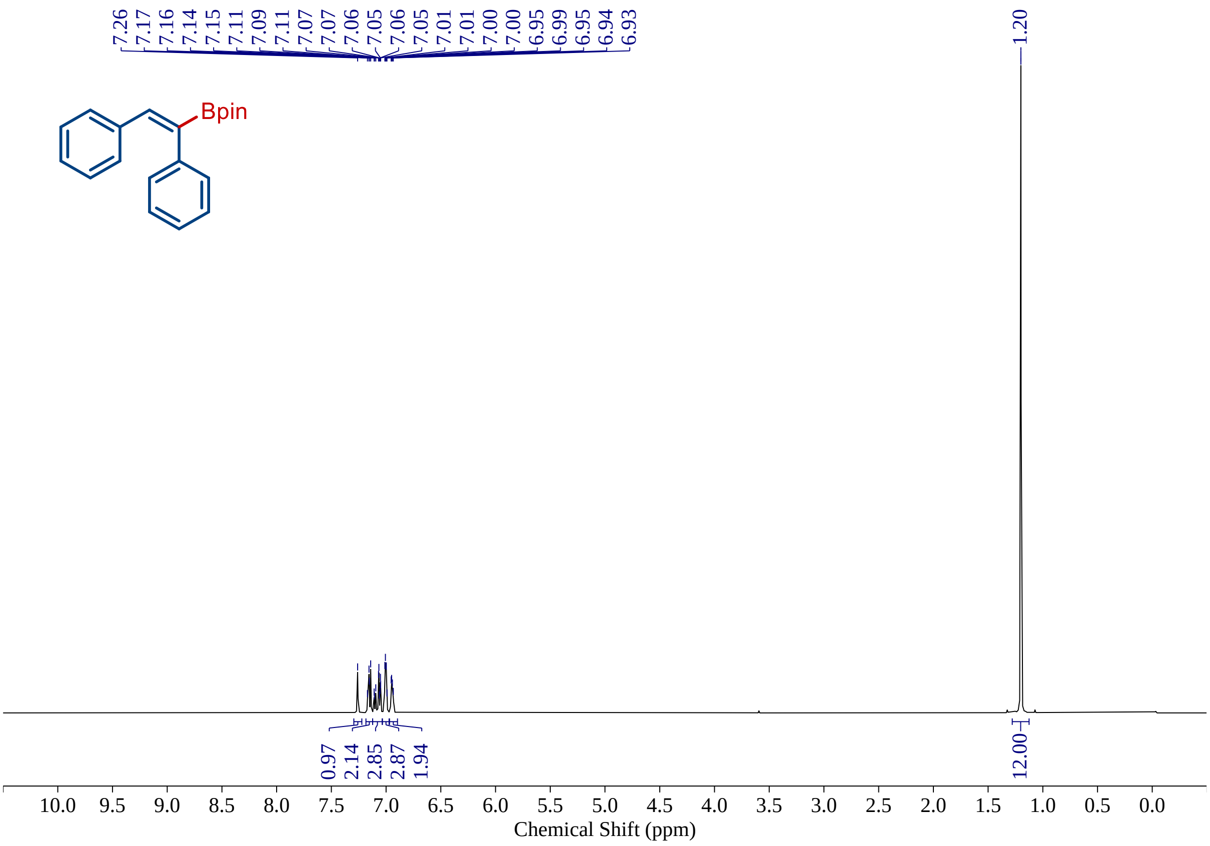

Figure S16. ^{1}H NMR (500 MHz, $CDCl_3$) Spectrum of Compound **11**.

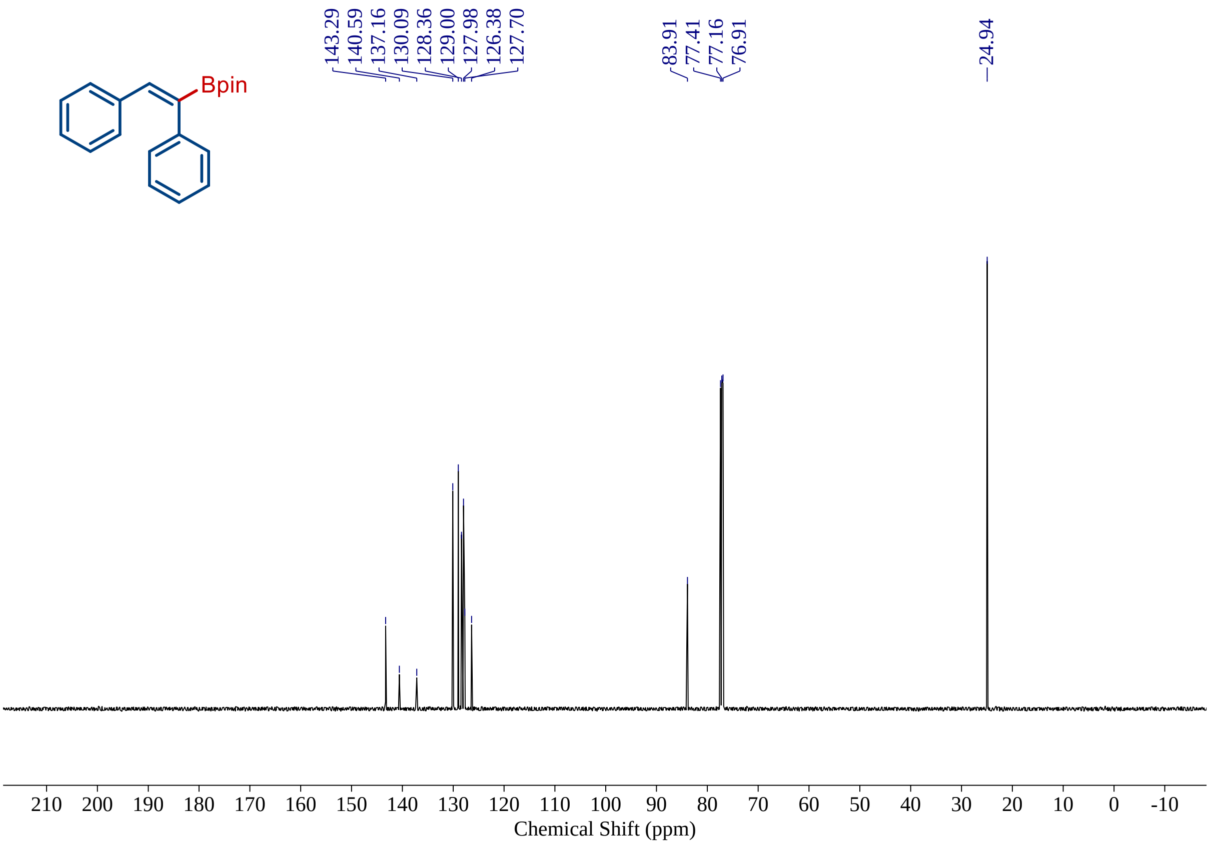

Figure S17. ^{13}C NMR (126 MHz, $CDCl_3$) Spectrum of Compound **11**.
